# MicroRNA Expression in Abdominal and Gluteal Adipose Tissue Is Associated with mRNA Expression Levels and Partly Genetically Driven

**DOI:** 10.1371/journal.pone.0027338

**Published:** 2011-11-15

**Authors:** Mattias Rantalainen, Blanca M. Herrera, George Nicholson, Rory Bowden, Quin F. Wills, Josine L. Min, Matt J. Neville, Amy Barrett, Maxine Allen, Nigel W. Rayner, Jan Fleckner, Mark I. McCarthy, Krina T. Zondervan, Fredrik Karpe, Chris C. Holmes, Cecilia M. Lindgren

**Affiliations:** 1 Department of Statistics, University of Oxford, Oxford, United Kingdom; 2 Wellcome Trust Centre for Human Genetics, University of Oxford, Oxford, United Kingdom; 3 Oxford Centre for Diabetes, Endocrinology and Metabolism, Churchill Hospital, Headington, Oxford, United Kingdom; 4 Novo Nordisk A/S, Måløv, Denmark; 5 NIHR Oxford Biomedical Research Centre, ORH Trust, Churchill Hospital, Oxford, United Kingdom; 6 MRC Mammalian Genetics Unit, MRC Harwell, Harwell, Oxford, United Kingdom; Wayne State University, United States of America

## Abstract

To understand how miRNAs contribute to the molecular phenotype of adipose tissues and related traits, we performed global miRNA expression profiling in subcutaneous abdominal and gluteal adipose tissue of 70 human subjects and characterised which miRNAs were differentially expressed between these tissues. We found that 12% of the miRNAs were significantly differentially expressed between abdominal and gluteal adipose tissue (FDR adjusted p<0.05) in the primary study, of which 59 replicated in a follow-up study of 40 additional subjects. Further, 14 miRNAs were found to be associated with metabolic syndrome case-control status in abdominal tissue and three of these replicated (primary study: FDR adjusted p<0.05, replication: p<0.05 and directionally consistent effect). Genome-wide genotyping was performed in the 70 subjects to enable miRNA expression quantitative trait loci (eQTL) analysis. Candidate miRNA eQTLs were followed-up in the additional 40 subjects and six significant, independent ***cis-***located miRNA eQTLs (primary study: p<0.001; replication: p<0.05 and directionally consistent effect) were identified. Finally, global mRNA expression profiling was performed in both tissues to enable association analysis between miRNA and target mRNA expression levels. We find 22% miRNAs in abdominal and 9% miRNAs in gluteal adipose tissue with expression levels significantly associated with the expression of corresponding target mRNAs (FDR adjusted p<0.05). Taken together, our results indicate a clear difference in the miRNA molecular phenotypic profile of abdominal and gluteal adipose tissue, that the expressions of some miRNAs are influenced by ***cis***-located genetic variants and that miRNAs are associated with expression levels of their predicted mRNA targets.

## Introduction

Different adipose depots have distinct endocrine and physiological properties [1], [2], [3]; increased amounts of abdominal adipose tissue are associated with an adverse metabolic risk while gluteal adipose tissue appears to have a relatively protective role with respect to type 2 diabetes (T2D), hypertension and dyslipidaemia [4], [5]. As the relative distribution of adipose tissue on the human body is related to the risk for general metabolic deregulation and increased morbidity, characterisation of the molecular phenotypes in different adipose depots is an important starting point when attempting to understand individual molecular mechanisms associated with adiposity and related disease risk. Further knowledge of tissue specific, disease-associated mechanisms may help in development of interventions, as well as preventive measures, to reduce risk of severe disease.

MicroRNAs (miRNAs) are short (19–22 nucleotides), evolutionarily conserved, non-coding RNA molecules, involved in gene regulatory functions. MiRNAs operate through a mechanism involving complementary sequence binding (of a seed region) to the 3′ UTR region of a target mRNA molecule. Formation of the miRNA:mRNA complex results in either increased degradation of the target mRNA molecule [6], or alternatively, inhibition of target mRNA translation [7], [8], [9]. Through these mechanisms, miRNAs have been predicted to affect the regulation of up to 30% of protein coding genes in mammals [8] and are consequently involved in regulating a broad set of cellular processes [10], [11]. Several examples of miRNA-mediated regulation in mammalian adipose tissue have been reported so far, including miRNA involvement in adipocyte differentiation [12], [13] and adipogenesis [14], [15]. Furthermore, the expression levels of several miRNAs have previously been reported to be associated with obesity, metabolic syndrome and T2D [16], [17], [18], [19]. The influence of genetic variants on miRNA expression has recently been reported in a study of miRNA expression in human fibroblasts [20], where 12 *cis* miRNA eQTLs were reported as significant, out of the 121 miRNAs tested (N = 180), motivating us to assess if genetic drivers of miRNA expression will also be present in adipose tissue.

Here, we studied global miRNA expression in gluteal and abdominal adipose tissues in 70 human subjects in the MolOBB study (41 healthy controls and 29 metabolic syndrome cases, see **Materials and Methods**), with the objective of characterising the global miRNA molecular phenotypic profile in these two adipose depots. To our knowledge, this is the first time a comprehensive miRNA characterisation has been carried out in human gluteal and abdominal adipose tissue depots. We have assessed whether miRNAs are differentially expressed between abdominal and gluteal adipose tissue and to what extent the expression of miRNAs are associated with metabolic syndrome in each tissue type. To determine the extent to which miRNA expression in adipose tissue is associated with predicted mRNA targets, indicating a potential a gene-regulatory activity of the miRNA, we have also carried out global mRNA expression profiling in both tissue types in the same set of subjects. Finally, all subject in the study were SNP genotyped and miRNA eQTL analysis was carried out, in order to assess whether miRNA expression levels are genetically driven.

## Results

### Differential miRNA expression between abdominal and gluteal adipose tissue

We profiled miRNA expression of 1,146 human miRNAs (based on miRBase 12.05 and additional in-house predictions by Illumina) in subcutaneous gluteal and abdominal adipose tissue from 70 human subjects, using the human Illumina miRNA BeadArray (version 2). A linear mixed-effects model was applied to model the tissue differential expression for each miRNA while correcting for relevant covariates, including metabolic syndrome case-control status (see **Materials and Methods**). We found 136 (12%) miRNAs to be significantly differentially expressed (FDR-adjusted p-value<0.05) between the gluteal and abdominal fat tissue samples in the primary study (**Table S1**). Of these, 61 (45%) had higher expression levels in gluteal fat, while 75 (55%) were expressed at higher levels in abdominal fat, indicating a clear difference in the molecular miRNA phenotype in each adipose depot. We also noted that the expression of 19 (14%) of these depot-specific miRNAs **(Table S1**) have previously been reported to be associated with adipose tissue development, obesity, T2D and metabolic disturbances [12], [13], [15], [21], [22], [23], [24], [25], [26], [27], [28], [29], [30], [31], [32]. Of these, nine miRNAs (*hsa-miR-326* [*21*] *, hsa-miR-211* [*22]*, [23**] *, hsa-miR-10b* [*32*] *, hsa-miR-365* [*32*] *, hsa-miR-10a* [*32*] *, hsa-miR-503* [*32*] , hsa-miR-335* [*30*] *, hsa-miR-331-3p* [*30*] *and has-miR-199a-5p* [*30*]) were expressed at higher levels in abdominal, rather than gluteal, adipose tissue **(Table S1)**. To confirm that miRNAs with tissue-differential expression did not have substantial metabolic syndrome case- or control group specific variability, the analysis was also carried out separately in each one of the case- and control groups and compared to the joint analysis. Results indicate a high degree of concordance between the joint analysis and analysis in case- and control groups separately (correlations of 0.93 and 0.98 were observed between the effect size estimates in the joint analysis and the separate analyses in case- and control groups respectively (**Figure S1**)).

**Figure 1:**
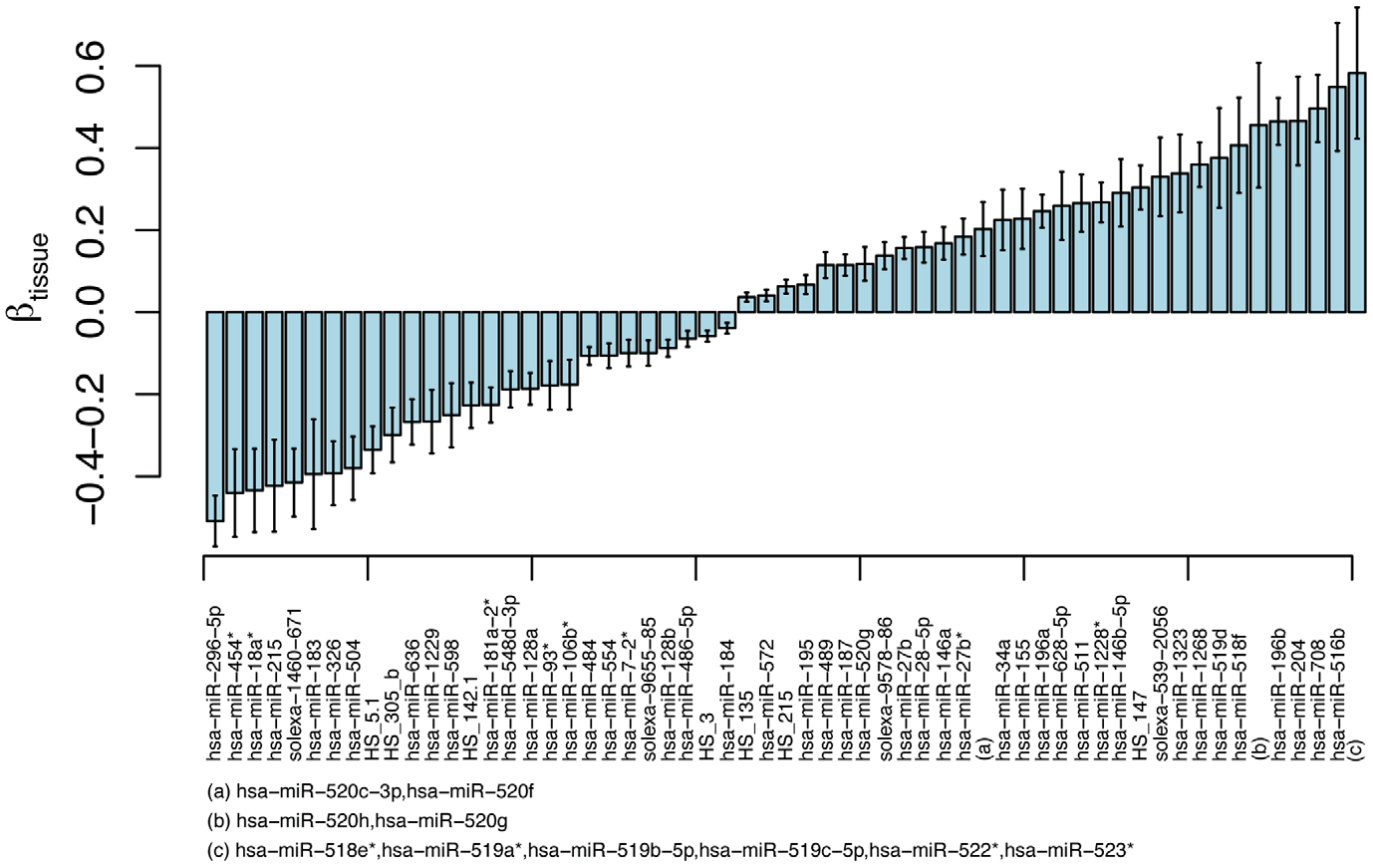
Tissue differential expression (the tissue-related fixed-effects coefficients (β)), of the 59 miRNA that were replicated (p-value<0.05) in the replication study. The coefficients (β) are sorted on effect-size, a positive value indicating higher expression in gluteal adipose tissue relative abdominal adipose tissue. Error-bars show the standard-error of β for each miRNA.

To replicate our results, we repeated the experiment and analysis in gluteal and abdominal adipose tissue from 40 additional human subjects (28 healthy controls and 12 metabolic syndrome cases), not part of the original study (see **Materials and Methods**), and only considered miRNAs that were found to be significant in the primary study. Of the 136 miRNAs found to be significantly differentially expressed in the primary study, 59 (44%) were also significantly differentially expressed in the replication study (p-value<0.05 and directionally consistent effect) (**Figure 1** **,** see **Table S1** for detailed results). When we compared the effect sizes across both the primary and replication studies, we observed a high degree of overall concordance (Pearson correlation coefficient = 0.86) between the coefficients (**Figure S2**), indicating a high level of agreement between the two studies, even among miRNAs that did not meet the p-value criterion for achieving replication. Seven of the miRNAs that were differentially expressed in both studies, have previously been reported to play a role in tissue development, obesity, T2D and metabolic disturbances (*hsa-miR-34a* [*23]*, [32**] *, hsa-miR-28-5p* [*32*] *, hsa-miR-27b* [*13]*, [15**] *, hsa-miR-326* [*21*] *, hsa-miR-204* [*22]*, [23**] *, hsa-miR-195* [*32*] *, hsa-miR-519d* [*29*]).

### miRNA expression associated with metabolic syndrome

To investigate the extent to which miRNA expression levels are associated with metabolic syndrome case-control status among the subjects in this study, we applied a similar model (see **Materials and Methods**) to that used in the analysis of tissue differential expression. The case-control analysis was performed separately for abdominal and gluteal adipose tissues. In abdominal tissue we found that 14 miRNAs were associated (FDR adjusted p-value<0.05) with metabolic syndrome case-control status (primary study). In the replication study three (21%) out of these 14 miRNAs were replicated (p-value<0.05 and directionally consistent effect), which is more than expected under the null, at the 0.05 level (p-value = 0.03 (one-sided binomial test)). The three replicated miRNAs, *hsa-miR-652*, *hsa-miR-1179*, hsa-miR-7-2*, all had lower expression levels in the case group. Furthermore, 13 out of 14 miRNAs also had coefficients that were directionally consistent across the primary and replication studies (**Figure S3**, **Table S2**). In gluteal adipose tissue, 14 miRNAs were differentially expressed (FDR adjusted p-value<0.05) between case and control groups in the primary study, but none of these replicated in the replication study. We did, however, observe directionally consistent effect estimates for 10 of the 14 miRNAs found to be differentially expressed in the primary study (**Figure S4**, **Table S3**). We note that the replication study had a lower proportion of metabolic syndrome cases due to sample availability (30% in comparison to 41% in the primary study).

### miRNA expression-Quantitative Trait Loci (eQTL) analysis

We then carried out miRNA eQTL analysis on the full set of miRNAs that were profiled, to assess whether genetic variation influences the expression level of miRNAs. We performed the eQTL analysis by modelling the effect of all *cis-*located SNPs on each of the target miRNAs, using a linear mixed-effects model, assuming an additive genetic effect and correcting for relevant covariates (see **Materials and Methods**). Abdominal and gluteal adipose tissue samples were considered separately in the miRNA eQTL analysis. We considered *cis-*SNPs located within an arbitrarily chosen region of 50 kilobases (kb) up-/down-stream from the miRNA sequence in the analysis. As in the analysis of tissue-specific differential miRNA expression (described above), we employed a likelihood ratio test to establish the ranking and significance of the genetic effect in each eQTL model (see **Materials and Methods**). These analyses revealed a modest excess of low p-values compared to the null distribution (see **Figure S5**). We carried forward 19 miRNA eQTLs in abdominal fat tissue (**Table S4)** and 11 in gluteal fat tissue (**Table S5**) for replication, using an arbitrary p-value threshold<0.001 (see **Materials and Methods**).

**Figure 2:**
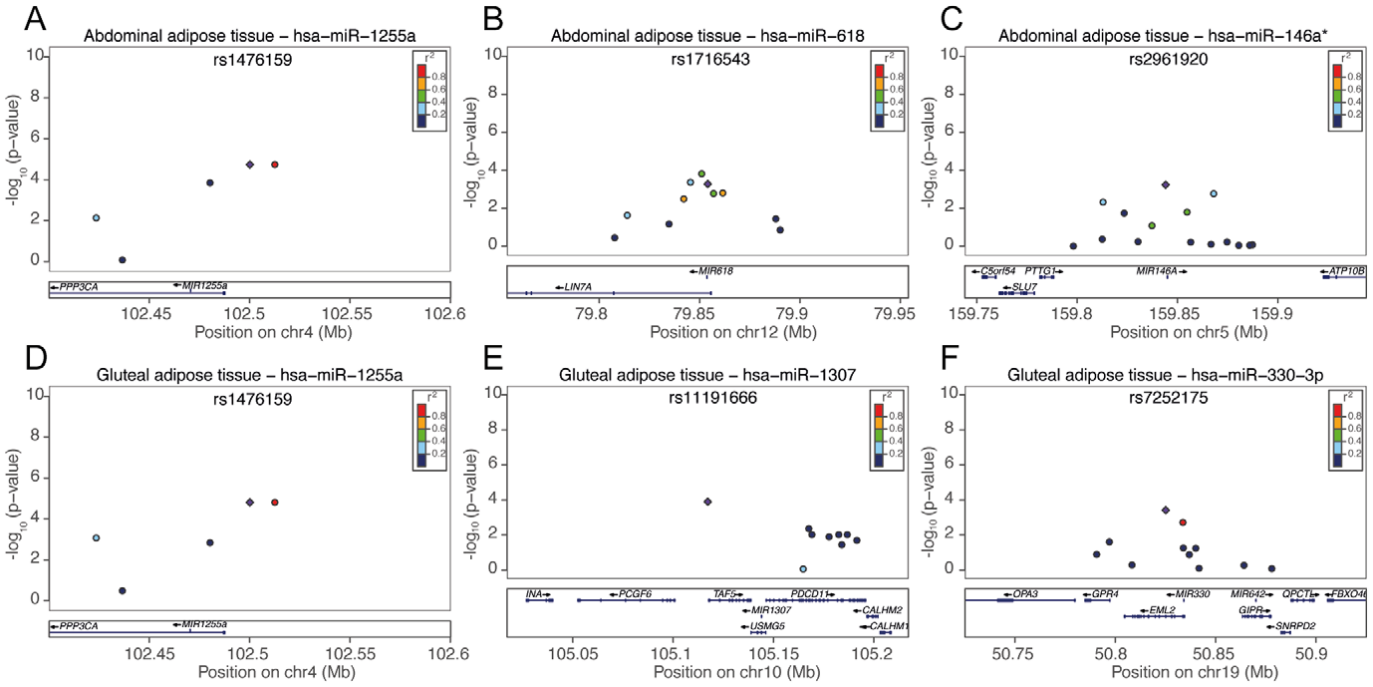
Locus plots for miRNA eQTLs that replicated. P-values from the primary study are shown in figure (criteria for replication: p-value<0.001 in the primary study and p-value<0.05 in the replication study with a directionally consistent effect). A) Abdominal adipose tissue *hsa-miR-1255a*:rs1822168 (p-value = 1.83E-05 in primary study and p-value = 9.91E-03 in replication study) B) Abdominal adipose tissue *hsa-miR-618*: rs1716543 (p-value = 5.34E-04 in primary study and p-value = 5.80E-03 in replication study) C) Abdominal adipose tissue hsa-miR-146a*:rs2961920 (p-value = 5.87E-04e in primary study and p-value = 6.45E-06 in replication study) D) Gluteal adipose tissue *hsa-miR-1255a*:rs1822168 (p-value = 1.56E-05 in primary study and p-value = 1.65E-04 in replication study), E) Gluteal adipose tissue *hsa-miR-1307*:rs11191666 (p-value = 1.27E-04 in primary study and p-value = 3.55E-04 in replication study), F) Gluteal adipose tissue *hsa-miR-330-3p*:rs7252175 (p-value = 3.85E-04 in primary study and p-value = 4.04E-02 in replication study).

**Figure 3:**
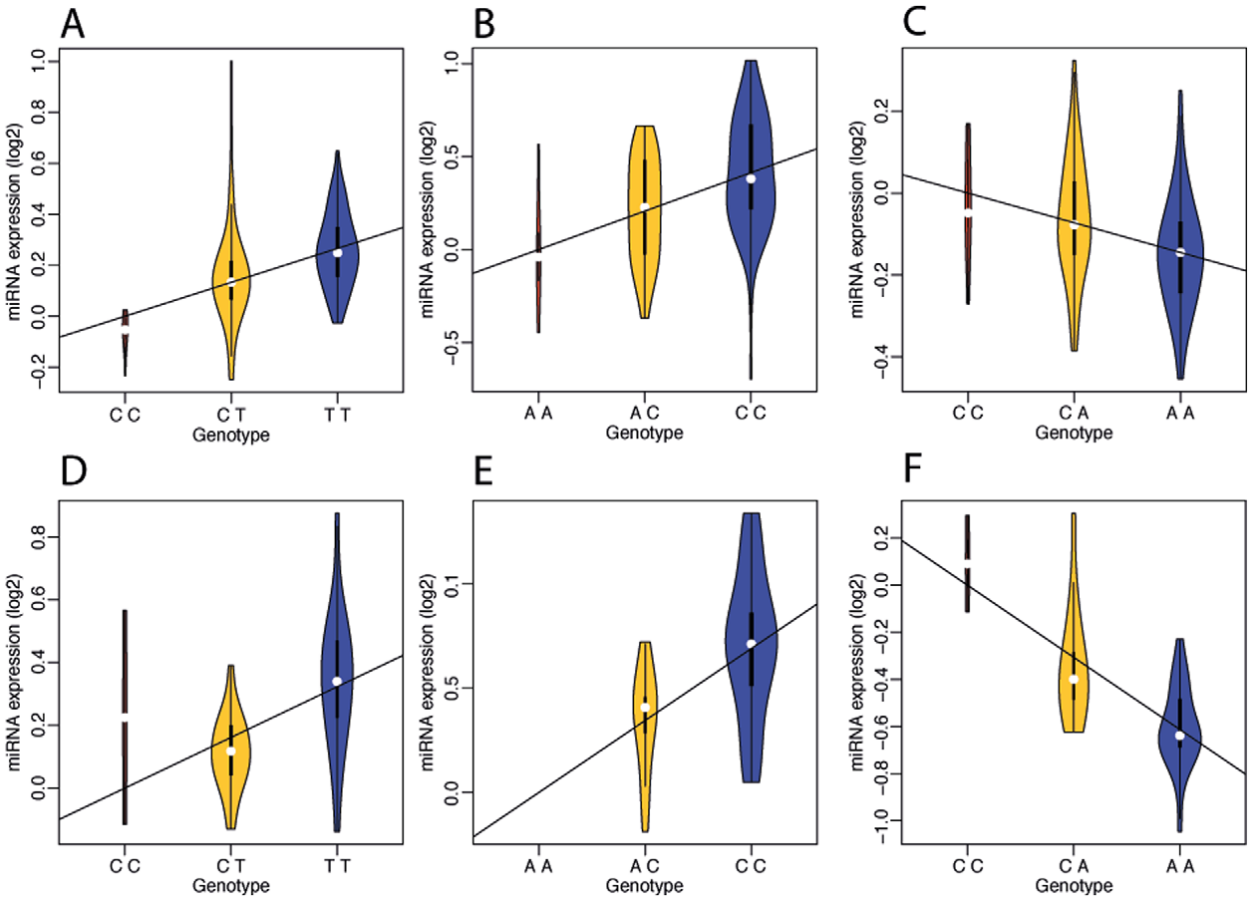
Genetic effects in replicated miRNA eQTLs in abdominal adipose tissue (see Materials and Methods section for criteria). The violin-plot represents the density of miRNA expression relating to the SNP effect for each genotype (variability relating to the other fixed effects was regressed out); the line represents the fixed-effect coefficient (*β*) relating to the SNP effect. A) Primary study *hsa-miR-1255a*:rs1822168 B) Primary study *hsa-miR-618*:rs1716543 C) Primary study hsa-miR-146a*:rs2961920 D) Replication study hsa-miR-1255a:rs1822168 E) Replication study *hsa-miR-618*:rs1716543. F) Replication study hsa-miR-146a*:rs2961920.

**Figure 4:**
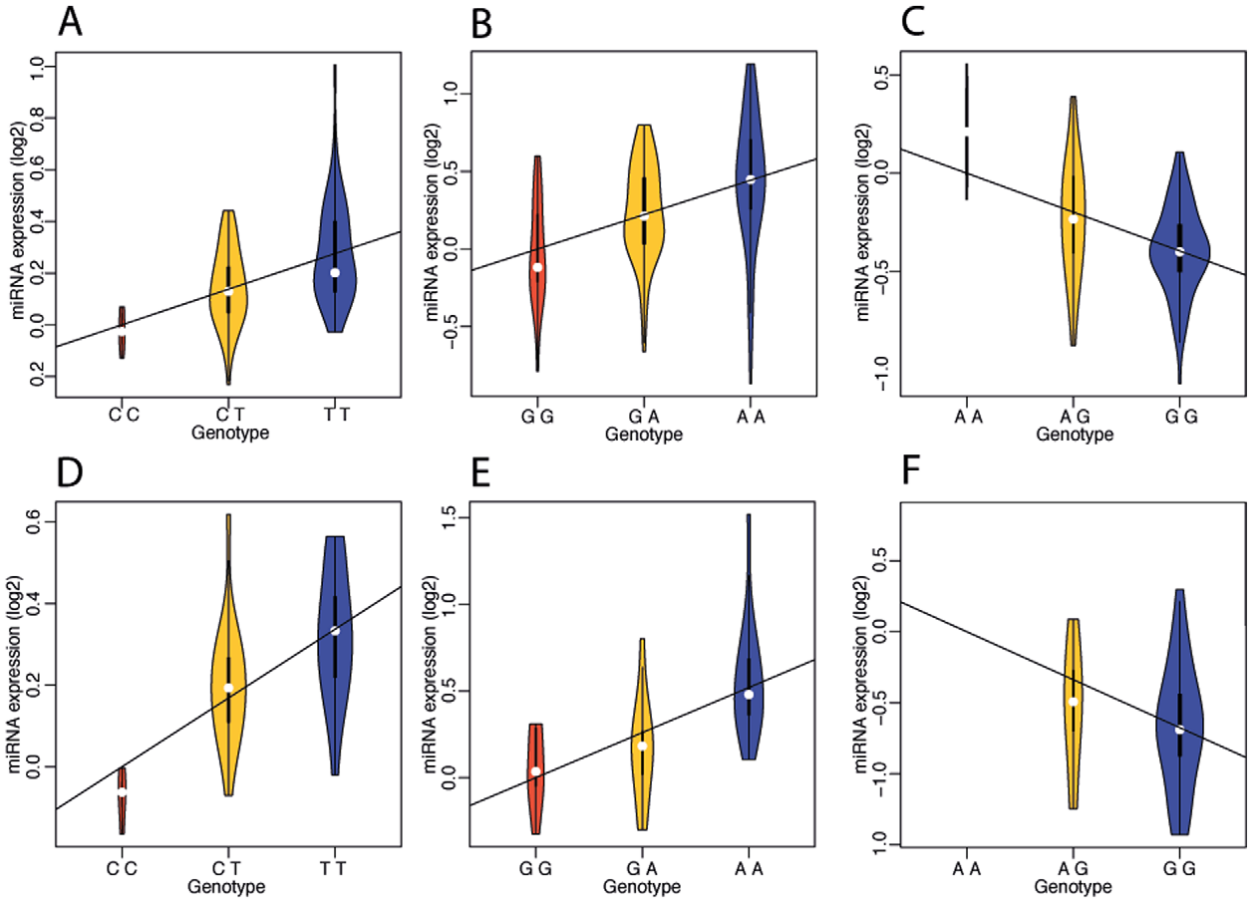
Genetic effects in replicated miRNA eQTLs in gluteal adipose tissue (see Materials and Methods section for criteria). The violin-plot represents the density of miRNA expression relating to the SNP effect for each genotype (variability relating to other fixed effects was regressed out); the line represents the fixed-effect coefficient (*β*) relating to the SNP effect. A) Primary study *hsa-miR-1255a*:rs1822168 B) Primary study *hsa-miR-1307*:rs11191666. C) Primary study *hsa-miR-330-3p*:rs7252175 D) Replication study *hsa-miR-1255a*:rs1822168. E) Follow up study *hsa-miR-1307*:rs11191666. F) Replication study *hsa-miR-330-3p*:rs7252175.

The candidate miRNA eQTLs were followed up in the replication cohort of 40 subjects, where three independent miRNA eQTL signals were replicated (p-value<0.05 and directionally consistent effect) in both abdominal adipose tissue (**Figure 2A–C** **,** **Figure 3** **,** **Table 1**) and gluteal adipose tissue (**Figure 2D–F** **,** **Figure 4** **,** **Table 2**). We note that *hsa-miR-1255a* has a significant eQTL signal in both abdominal and gluteal adipose tissues (**Table 1** **and** **2**) and was not found to be differentially expressed between the depots (**Table S1**).

**Table 1:** Replicating miRNA eQTLs in abdominal adipose tissue.

					Primary study				Replication study		
miRNA^a^	SNP(rs)^b^	Chr^c^	SNP (Position)^d^	Effect allele^e^	β_snp_ f	s.e.(β_snp_ g)	p-value^h^	FDR adjustedp-value^i^	β_snp_ (study2)^j^	s.e(β_snp_) (study2)^k^	p-value (study2)^l^
hsa-miR-1255a	rs1822168	4	102512567	C	0.133	0.029	1.83E-05	0.081	0.161	0.060	9.91E-03
hsa-miR-618	rs1716543	12	79854071	A	0.207	0.057	5.34E-04	0.316	0.345	0.116	5.80E-03
hsa-miR-146a*	rs2961920	5	159844084	C	−0.073	0.021	5.87E-04	0.326	−0.306	0.058	6.45E-06

a miRNA name,

b rs identifier for each SNP,

c chromosome number,

d genomic location of SNP,

e effect allele,

f coefficient for the SNP effect in the primary study,

g standard error for the SNP coefficient in the primary study,

h p-value for the SNP effect in the primary study,

i FDR adjusted p-value for the SNP effect in the primary study,

j coefficient for the SNP effect in the replication study,

k standard error for the SNP coefficient in the replication study,

l p-value for the SNP effect in the replication study.

**Table 2:** Replicating miRNA eQTLs in gluteal adipose tissue.

					Primary study				Replication study		
miRNA^a^	SNP(rs)^b^	Chr^c^	SNP (Position)^d^	Effect allele^e^	β_snp_ f	s.e.(β_snp_ g)	p-value^h^	FDR adjustedp-value^i^	β_snp_ (study2)^j^	s.e(β_snp_) (study2)^k^	p-value (study2)^l^
hsa-miR-1255a	rs1822168	4	102512567	C	0.138	0.029	1.56E-05	0.069	0.169	0.036	1.65E-04
hsa-miR-1307	rs11191666	10	105117268	G	0.221	0.054	1.27E-04	0.282	0.260	0.065	3.55E-04
hsa-miR-330-3p	rs7252175	19	50825096	A	−0.198	0.053	3.85E-04	0.595	−0.338	0.160	4.04E-02

a miRNA name,

b rs identifier for each SNP,

c chromosome number,

d genomic location of SNP,

e effect allele,

f coefficient for the SNP effect in the primary study,

g standard error for the SNP coefficient in the primary study,

h p-value for the SNP effect in the primary study,

i FDR adjusted p-value for the SNP effect in the primary study,

j coefficient for the SNP effect in the replication study,

k standard error for the SNP coefficient in the replication study,

l p-value for the SNP effect in the replication study.

To investigate whether any of the five miRNA eQTL SNPs were directly associated with obesity phenotypes (body mass index and waist hip ratio corrected for BMI), we looked up association p-values in a genome-wide association study with 113,636 subjects from the GIANT consortium) [33], [34]. We found that none of the individual miRNA eQTL-related SNPs had a significant (p-value<0.05) association with either of the two obesity phenotypes (**Table S6**), providing no direct evidence that the miRNA eQTL SNPs are associated with either of these particular phenotypes.

### Association between miRNA and target mRNA expression

An anti-correlated association between the expression level of a miRNA and its predicted mRNA targets may indicate a potential mRNA degrading effect of the miRNA [35]. To investigate whether miRNAs were associated with their mRNA targets in adipose tissue, we applied a gene set enrichment (GSE) test [36]. Global mRNA expression was profiled using the Affymetrix human GeneChip HGU133 Plus 2.0 array (see **Materials and Methods**) and mRNA expression profiles from 50 of the abdominal adipose samples and 55 of the gluteal adipose samples were available for integrated miRNA-mRNA analysis.

**Figure 5:**
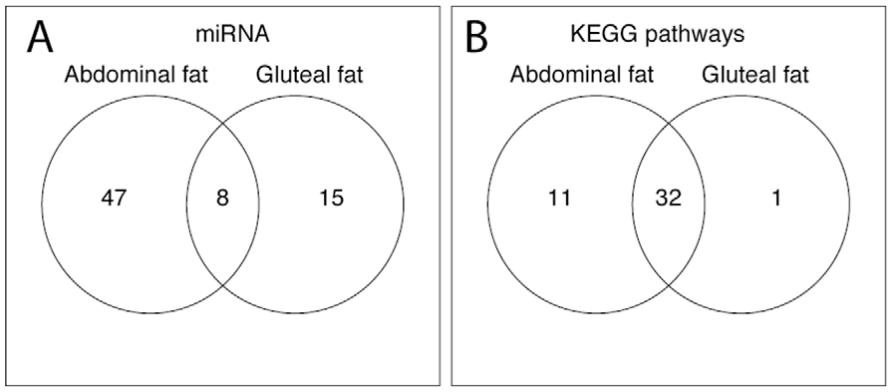
(A) Venn diagram showing the number of common and unique miRNAs with significant association to their target mRNAs in each adipose tissue type. (B) Venn diagram showing the number of common and unique KEGG terms in each adipose tissue type.

For each miRNA with conserved predicted mRNA targets (defined by TargetScan [37]), a one-sided GSE test was carried out to test if the miRNA had a significant anti-correlated association with its set of predicted mRNA targets. The GSE test was based on statistics from association tests between a particular miRNA and all of the mRNAs, one at a time, using a linear fixed-effects model adjusted for relevant covariates, with the miRNA as the predictor and one mRNA as the response (see **Materials and Methods**). In the GSE analysis, we test whether there was an enrichment of highly ranked test statistics within the set of predicted mRNA targets of the miRNA being tested, with the null hypothesis that the predicted target set was randomly chosen (see **Materials and Methods**). The analysis included 248 profiled miRNAs with conserved predicted mRNA targets, since these are thought to be more likely to have a gene-regulatory function [38]. Thus, we performed 248 GSE analyses. In abdominal adipose tissue 55 out of 248 (22%) miRNAs were found to have a significant (FDR adjusted p-value<0.05) association with their predicted conserved target mRNAs (**Table S7**). In gluteal adipose tissue, the same analyses revealed that 23 out of 248 (9%) of miRNAs had a significant association with their predicted conserved target mRNAs (**Table S8**). Eight of the miRNAs that showed significant associations with their target mRNAs, were common between the two tissue types: *hsa-miR-181a, hsa-miR-186, hsa-miR-30a, hsa-miR-141, hsa-miR-30d, hsa-miR-590-3p, hsa-miR-128 and hsa-miR-340* (**Figure 5A**).

### Functional analyses based on miRNA-associated mRNAs in abdominal and gluteal adipose tissue

To determine the general functional characteristics of the miRNA-related activity in each adipose tissue type, we looked at miRNAs that were significantly associated with their mRNA targets, and tested whether or not there was an enrichment of specific KEGG [39] terms in each tissue. The analyses included the 248 miRNAs with predicted conserved mRNA targets based on Target Scan, using the same set of miRNAs included in the miRNA-mRNA GSE analysis (see previous section). For each miRNA that had a significant association with its set of mRNA targets (based on the gene set enrichment test), the KEGG terms were extracted for all of its mRNA targets. KEGG terms were aggregated across the significant miRNA-mRNA sets separately for each tissue. We then applied Fisher's exact test to assess if any of the KEGG terms were enriched in these candidate sets of KEGG terms (see **Materials and Methods**). The analysis revealed an enrichment of 43 KEGG terms in abdominal adipose tissue (**Table S9**), and 33 in gluteal adipose tissue (**Table S10**) (FDR adjusted p-value<0.05). We found that 32 of the KEGG terms were common between the two tissues (**Figure 5B**). Several of these KEGG terms were related to obesity, adipocyte differentiation and metabolic dysregulation; including MAPK signaling pathway, Insulin signaling pathway, Type II diabetes mellitus and Adipocytokine signalling pathway, suggesting relevant functionality.

## Discussion

Adiposity and body fat distribution are both heritable traits, while the heritability of body fat distribution is distinct from that of overall adiposity [40], [41], [42], [43]. Body fat distribution is divided into two general types: android (upper body or ‘apple’ shape) and gynoid (lower body or ‘pear’ shape), where an android fat distribution is more commonly associated with adverse metabolic outcomes [44]. Recent evidence suggests that these differences are driven by adipocytes at different fat depots, which have unique physiological functions and metabolism [45], [46], [47]. As the relative distribution of adipose tissue on the human body is related to the risk of general metabolic dysregulation, characterisation of the molecular phenotypes in the different adipose tissue depots is an essential step towards uncovering the individual molecular mechanisms associated with adiposity and related disease risk.

Earlier studies have reported that miRNA expression is important in both adipose tissue development and metabolism [16], [17], [18], [19], [48]. In this study we have profiled global miRNA expression in two body fat depots and investigated whether miRNAs contribute to the overall molecular phenotype in gluteal and abdominal adipose deposits. Our analysis detected significant differential miRNA expression between these two fat depots. Interestingly, a proportion of the miRNAs found to be significantly differentially expressed in our study, had previously been reported to play a role in adiposity, adipocyte development/differentiation and other metabolic disturbances; further highlighting their importance (**Table S1**) [12], [13], [21], [22], [23], [24], [25], [26], [27], [28], [29], [30], [31], [32]. Our analysis also revealed three miRNAs (*hsa-miR-652*, *hsa-miR-1179*, hsa-miR-7-2*) differentially expressed between control and metabolic syndrome groups in abdominal adipose tissue. *Hsa-miR-652* has only three conserved mRNA targets included in the Target Scan database: *ISL1*, *ACVR2B* and *PSKH1*. ISL1 is a transcription factor of the LIM/homeodomain family, involved in regulating expression of the insulin gene in islet cells [49] and previously noted to be differentially expressed between visceral and subcutaneous fat and negatively correlated with BMI [50]. Taken together, our results, supported by recent publications, reflect an important role of miRNAs in contributing to the general molecular phenotypic profile in each one of these two adipose tissues.

Genetic variants affecting miRNA expression suggest a possible mechanism through which genetic variants can influence downstream molecular- or phenotypic traits, and emphasise the need to characterise the extent to which miRNA expression levels are associated with common genetic variants in adipose tissue. For example, SNPs may exert an effect on miRNA expression, which may subsequently affect mRNA expression, and ultimately, phenotypic traits. MiRNA eQTL analysis allows us to assess the first part of this mode of action. We found that several miRNAs do indeed have significant eQTLs, suggesting, at least for a subset of miRNAs, that there is evidence of genetic variation controlling miRNA expression levels in human adipose tissue. Our results are in line with recent data from a study of human fibroblasts [20], as well as rodent models of T2D, where the expression of ∼10% of miRNAs were reported to be under genetic control [23]. We note that the relatively high FDR adjusted p-values (up to 0.33 in abdominal adipose tissue, and up to 0.6 in gluteal adipose tissue) (**Table 1**, **Table 2**) in the eQTL analysis in the primary study are in line with eQTL results presented in [20], where a FDR level of 0.5 was reported. Comparing the set of *cis* miRNA eQTL candidates in the primary study with the 12 *cis* miRNA eQTL reported in human fibroblasts [20], shows that in these two studies the detected miRNA eQTLs were not common. This may suggest that miRNAs expression is tissue-type specific, which our results also indicate when comparing miRNA expression between adipose tissue depots.

Since our miRNA analyses revealed differential miRNA expression associated with adipose tissue depots, metabolic syndrome and specific *cis*-located genetic variants, we were interested in assessing if miRNA variability had any association with target mRNA expression levels. Such an association, particularly if anti-correlated, might indicate an active regulatory role of miRNA on their target mRNAs, providing an indication of down-stream effects related to miRNA expression variability. Investigation of associations between miRNA and their predicted conserved mRNA targets revealed that a proportion of miRNAs had a significant effect on their target mRNAs in both abdominal and gluteal adipose tissue. Finally, we assessed the functional role of miRNA activity in adipose tissue by means of an enrichment analysis of KEGG terms in each tissue type. We found that a number of KEGG terms were common between the two tissue types, including MAPK signaling pathway, Insulin signaling pathway, Type II diabetes mellitus and Adipocytokine signalling pathway. We also noted that a proportion of the significant KEGG terms indicated pathways that are potentially relevant for T2D, obesity and related metabolic disorders in these adipose tissue depots (**Table S9** and **S10**).

Few of the miRNAs found significant in at least one of the four analyses (i.e. association with metabolic syndrome case-control, with *cis-*located genetic variants, with predicted target mRNAs or with tissue type) were found to be common across analyses (**Figure S6**). In abdominal tissue, hsa-miR-520c-3p/520f, hsa-miR-519d and hsa-miR-183 were found to be associated with their target mRNAs, as well as being tissue differentially expressed. Hsa-miR-7-2* was found to be significantly associated with case-control status in abdominal tissue and also tissue-differentially expressed. In gluteal tissue, hsa-miR-27b, hsa-miR-196b and hsa-miR-28-5p were found to be both associated with their target mRNAs, as well as being tissue differentially expressed. We note that few miRNAs were found to be significant in multiple analyses, although this might be explained by the fact that the current study has limited power to detect miRNA associations with metabolic syndrome case-control status and *cis-* located genetic variants (eQTLs). Another potential reason for finding only a few miRNAs that were significantly associated with mRNAs, is that only a subset (248) of the miRNAs that were profiled have predicted conserved mRNA targets. Thus, the miRNA-mRNA analysis is dependent on the accuracy and limitations of the target prediction algorithms currently available. Furthermore, we note that the three significant miRNA eQTLs detected in each tissue were not found to be associated with metabolic syndrome case-control status. However, these miRNAs eQTLs may well be associated with other (obesity-related) traits not included in the current study, which would be of interest to investigate in future studies.

Despite limitations in respect to sample sizes and statistical power, the current results have revealed significant miRNA activity in these body fat depots. Hence, these results should encourage further studies with larger sample sizes and increased power, necessary to detect additional miRNA eQTLs in adipose tissue, as well as for detecting associations between miRNA eQTLs and relevant obefsity-related phenotypes. The results from the miRNA-mRNA association analysis are also encouraging and highlight the imfportance of performing global profiling of both miRNA and mRNA expression within the same study, in order to determine the extent to which these two molecular phenotypes are associated.

Our results indicate that there is a clear difference in the miRNA molecular phenotypic profile of gluteal and abdominal adipose tissue and that miRNA expression is at least partly driven by effects related to common genetic variation. Our results also suggest an association between miRNA and mRNA expression levels, indicating that miRNAs may play an active role in gene regulation in adipose tissue. The exact mechanisms through which miRNAs act are still elusive and future work would need to include detailed functional studies (for the miRNAs where this is not already available), as well as proteomic studies, to find evidence of the implications of miRNA expression changes on the related protein abundances in adipose tissue.

## Materials and Methods

### Ethics statement

The study has received ethical approval from National Health Service (NHS), National Research Ethics Service, Oxfordshire REC C (REC reference: 08/H0606/107). Informed consent in writing was obtained from all participants involved in the study.

### Study design and experimental material and methods

#### Sample and subject information – primary study

Tissue samples from 70 human subjects (40 male and 30 female) belonging to either a healthy control group (N = 41, 24 male, 17 female) or the metabolic syndrome case group (N = 29, 16 male, 13 female) were collected from the Oxford Biobank [51]. Metabolic syndrome status was assigned based upon the International Diabetes Foundation (IDF) criteria [52]. Control subjects were selected to be discordant from the metabolic syndrome cases. Adipose tissue samples from abdominal and gluteal adipose tissues were collected from all subjects. DNA and total RNA were extracted from each subject and sample.

#### Sample and subject information – replication study

To validate results from the primary study, gluteal and abdominal adipose tissue samples from 40 additional subjects (20 male, 20 female) were collected from the Oxford Biobank. Among the females 5 subjects were metabolic syndrome cases (3 IDF), and 15 were controls. Among the males 7 subjects were metabolic syndrome cases (3 IDF), and 13 were controls. A logistic regression model for case-control status, estimated on relevant clinical phenotypes (body mass index, blood high-density lipoprotein level, blood triglyceride level, blood fasting glucose level, waist circumference, diastolic blood pressure, systolic blood pressure) in the primary data set, was used to predict which of the available subjects in the Oxford Biobank that were most similar to the cases and controls in the primary study, with the aim of establishing a relevant and as similar as possible, replication study. Validation cohort metabolic syndrome subjects consisted of a set of cases fulfilling the formal IDF criteria, as well as a set of cases that were predicted as cases through the logistic regression model. As the main focus of this study is on tissue related effects, the subjects in the replication study are to be considered as relatively well matched to the primary study subjects.

#### Single Nucleotide Polymorphism (SNP) genotyping – primary study

DNA was extracted from each subject using GeneCatcher™ (Invitrogen Life Technologies, Carlsbad, USA) using manufacturers protocol before being genotyped on the Illumina 317 k Beadchip platform (Illumina Inc., San Diego, CA, USA).

#### Single Nucleotide Polymorphism (SNP) genotyping – replication study

Genotyping was performed using the Sequenom iPLEX™ assays with the mass spectrometry based MassARRAY™ platform for genotype detection (Sequenom, San Diego, USA), according to manufacturer's protocol.

#### miRNA expression profiling – primary study

Total RNA was extracted from homogenized adipose tissue samples from each subject using TRI Reagent (Sigma, Gillingham, UK) in accordance with manufacturer's procedure. RNA quality was assessed using a spectrophotometer (NanoDrop, Labtech International, UK) and the Bioanalyzer 2100 (Agilent, South Queensferry, West Lothian, UK). MiRNA expression profiling was carried out using the Illumina miRNA BeadArray platform (version 2), 96 sample universal array matrix format (MI-102-1196). The Illumina miRNA BeadArray platform includes probes against 859 known human miRNA sequences and 287 predicted miRNA sequences. Briefly, 400 ng of miRNA were used per sample, samples were polyadenylated, converted to biotinylated cDNA and hybridized to the BeadArray, and then a universal PCR amplification was performed resulting in fluorescently labelled product labelled by miRNA specific oligo (MSO) molecules. Arrays were then scanned using the BeadArray Reader (Illumina, San Diego, US). Samples were measured in technical duplicates (with a few in triplicates). Due to a small number of failed assays, data are available from 69 abdominal adipose samples (140 arrays including technical duplicates) and 66 gluteal adipose samples (134 arrays including technical duplicates). In total, data from 70 subjects and two tissue types, measured on 274 arrays, were available for further analysis in the primary study.

#### miRNA expression profiling – replication study

In total, data were collected from 40 subjects and both abdominal and gluteal adipose tissue, 13 of the 80 samples were measured in duplicates, resulting in data from 93 arrays available for further analysis in the replication study. 500 ng of total RNA from each sample were labelled and hybridized on Universal-12 Illumina BeadChip (Cat. No. # 11288011), according to the manufacturer's recommendations (Illumina microRNA expression profiling assay for BeadChips). BeadChips were scanned with the Illumina BeadArray scanner accordingly and images imported into GenomeStudio version 1.6.0 (illumina) to extract the raw data for further analysis.

#### mRNA expression profiling

Total RNA was extracted with TRIreagent (SIGMA-ALDRICH) from fat biopsies. RNA expression was analyzed on the Affymetrix human GeneChip HGU133 Plus 2.0 array (Affymetrix) covering over 47,000 transcripts genome-wide. Labelled RNA was hybridized onto Affymetrix arrays, washed, stained and scanned for fluorescence intensity corresponding to gene expression level.

### Data preprocessing

All data analysis was carried out using R version 2.9.0 [53] unless otherwise stated.

#### miRNA expression data preprocessing

Data was imported into R using the beadarray package [54]. Data was background corrected by subtracting the background intensity from the foreground levels and bead summary data was calculated using the Illumina method. Data were thereafter quantile normalised between arrays, log_2_ transformed and expression intensities were extracted. Normalisation was done separately for abdominal and gluteal adipose tissue samples for miRNA eQTL analysis, while in the case of tissue-differential expression analysis, all data were normalised together. Probes were excluded from further analysis if the 75^th^ percentile of expression level in either tissue was lower than the 25^th^ percentile of array control probes, resulting in 1131 (out of 1146) probes included for further analysis. Outliers were detected using Principal Component Analysis (PCA) and the Hotelling T^2^ test on the first 3 PCA score components; outliers were defined as being outside the 0.99 confidence interval in the score space. One array was defined as an outlier by PCA, two additional arrays were considered as outliers due to substantially higher overall background and foreground signals than other arrays, in total three arrays were excluded from further analysis. Technical reproducibility was assessed by calculating the correlation between duplicate array profiles with results indicating a high degree of concordance (Figure S7).

#### mRNA expression data preprocessing

mRNA expression data was collected on the Affymetrix human GeneChip HGU133 Plus 2.0 array and normalized using the RMA method [55] without background correction (i.e. quantile normalization followed by robust probe-set summarization). All expression data (from all abdominal and gluteal samples) were preprocessed together. Publicly available custom chip-definition files (CDFs) were downloaded (version 9) (http://brainarray.mbni.med.umich.edu/Brainarray/Database/CustomCDF/CDF_download.asp) and used to group probes into sets, each set corresponding to an Ensembl-annotated gene, resulting 17,209 such genes represented in the array data. See Dai *et al* [56] for a description of how these CDFs were created, along with a comparison of their properties with the CDFs produced by Affymetrix.

#### Single nucleotide polymorphism data preprocessing – primary study

Samples were genotyped using the Illumina 317 k Beadchip platform. One sample was removed due to non-European ancestry. SNPs were excluded if MAF<1% or if genotyping success rate was <95% and MAF >5%, and if genotyping success rate was <99% and MAF <5%. Hardy-Weinberg equilibrium was calculated by combining all unrelated and SNPs were removed if HWE p-value was <0.0001. In total, 69 samples were successfully genotyped for 302765 SNPs.

#### Single nucleotide polymorphism data preprocessing – replication study

Completely failing assays (<25% success rate) were first removed. Thereafter samples with <85% success rate were removed. Hardy-Weinberg equilibrium was calculated, and SNPs with HWE p-value <0.001 or success rate <85% were excluded from further analysis.

### Statistical analysis

#### miRNA eQTL analysis

eQTL analysis was performed for miRNAs located on chromosome 1–22. We consider *cis*-eQTLs in our analysis, *cis*-located SNP variants were defined as +/− 50 kilo-bases from miRNA genomic location. 729 miRNAs have at least one *cis*-located SNP, in total 9346 miRNA eQTL models were evaluated. The eQTLs were modelled using a linear mixed-effects model: *y_ij_* = *µ*+β*snp_i_+*τ_gender(i)_*+*η_caseControl(i)_*+*α_batch(i,j)_*+γ*age_i_+*κ_i_*+*ε_ij_*. Subjects are indexed by *i* ∈ {1…69}, aliquots are indexed by *j* ∈ {I,2}. *y_ij_* is the miRNA expression level of one of the miRNA probe for subject *i* and aliquot *j*. *µ* is overall mean expression level. *β* is a fixed effect representing the genetic effect of the SNP variant, where *snp_i_* is a function mapping individual *i* to the number of copies of the reference allele {0, 1, 2}. τ_gender(i)_ is a fixed effect corresponding to gender for subject *i,* where *gender(⋅)* is a function mapping individual *i* to gender ∈ {male, female}. η_caseControl(i)_ is a fixed effect representing case/control status of subject *i,* where *caseControl(⋅)* is a function mapping individual *i* to it's case/control status for metabolic syndrome ∈ {case, control}, γ is a fixed effect incorporating the effect of age, α_batch(i,j)_ is a fixed effect included to accommodate an experimental batch effect (96-well plate format), where the function *batch(⋅)* is a function mapping each sample to a batch (plate) ∈ {1,2,3}. The κ_I_ are independently Gaussian distributed with mean zero and variance σ^2^_κ,_ for subject *i* representing inter individual variability. ε_ij_ is the residual error term incorporating technical (aliquot) variability, assumed to be independently Gaussian distributed with mean zero and variance σ^2^_ε_. The model was fitted in R, using the lme4 package[57] and the *lmer()* function, using maximum-likelihood. A likelihood ratio test was applied to assess the significance of the SNP effect (*β*), which is the parameter of main interest in the eQTL analysis. The p-value of the SNP effect in each eQTL model was calculated using a likelihood ratio (LR) test with the D = −2*log(LR) as the test statistic, which can be approximated by a Chi-square distribution with one degree of freedom. The p-values were adjusted for multiple testing by FDR correction [58]. In the replication study the batch effect was omitted from the model due to change of Illumina platform from 96-array format to 12-array format with no apparent batch differences present. For the eQTL analysis results were considered to be replicating in the replication study if the fixed effect of interest (*β*) had a p-value<0.05 and consistent direction of the effect. If quality control criteria for SNP genotyping of particular SNPs were not met in the replication study, a proxy - SNP were instead reported with better genotyping quality.

#### Tissue-differential expression analysis

A linear mixed-effects model was fitted and evaluated for differential expression between miRNA expression in gluteal and abdominal tissues: *y_ijk_* = *µ*+*β_tissue(k)_*+*τ_gender(i)_*+*η_caseControl(i)_*+*α_batch(i,j)_*+γ*age_i_+λ_i_+*κ_ik_*+*ε_ijk_*. Subjects are indexed by *i* ∈ {1…70}, aliquots are indexed by *j* ∈ {1,2} and tissue type is indexed by *k* ∈ {1,2}. *y_ijk_* is the miRNA expression level of one of the miRNA probes for subject *i*, aliquot *j* and tissue *k*. *µ* is overall mean expression level. *β_tissue(k)_* is a fixed effect representing the tissue effect for adipose tissue type ∈ {abdominal, gluteal}. τ_gender(i)_ is a fixed effect corresponding to gender for subject *i,* where *gender(⋅)* is a function mapping individual *i* to it's gender ∈ {male, female}. η_caseControl(i)_ is a fixed effect representing case/control status of subject *i,* where *caseControl(⋅)* is a function mapping individual *i* to it's case/control status for metabolic syndrome ∈ {case, control}, γ is a fixed effect incorporating the effect of age, α_batch(i,j)_ is a fixed effect included to accommodate an experimental batch effect (96-well plate format), where the function *batch(⋅)* is a function mapping each sample to a batch (plate) ∈ {1,2,3}. The *λ_i_* are independently Gaussian distributed with mean zero and variance σ^2^_λ,_ for subject *i,* representing inter-individual variability across tissues. *κ*_ik_ are independently Gaussian distributed with mean zero and variance σ^2^_κ,_ for subject *i* and tissue *k,* representing within-individual variability. ε_ijk_ is the residual error term, incorporating technical (aliquot) variability, assumed to be independently Gaussian distributed with mean zero and variance σ^2^_ε_. The model was fitted in R, using the lme4 package[57] and the *lmer()* function, using maximum-likelihood. A likelihood ratio test was applied to assess the significance of the tissue effect (*β*), which is the parameter of main interest in the tissue differential expression analysis. The p-value of the tissue effect in each model was calculated using a likelihood ratio (LR) test with the D = −2*log(LR) as the test statistic, which can be approximated by a Chi-square distribution with one degree of freedom. The p-values were adjusted for multiple testing by FDR correction [58]. In the replication study the batch effect was omitted from the model due to change of Illumina platform from 96-array format to 12-array format with no apparent batch differences present. For the tissue-differential analysis results were considered to be replicating if the fixed effect of interest (*β*) had a p-value<0.05 and consistent direction of the effect.

#### Metabolic syndrome case-control analysis

Metabolic syndrome case-control analysis was performed using a linear mixed-effects model: *y_ij_* = *µ*+*τ_gender(i)_*+*η_caseControl(i)_*+*α_batch(i,j)_*+γ*age_i_+*κ_i_*+*ε_ij_*. The analysis was performed separately for each tissue. Subjects are indexed by *i* ∈ {1…70}, aliquots are indexed by *j* ∈ {I,2}. *y_ij_* is the miRNA expression level of one of the miRNA probe for subject *i* and aliquot *j*. *µ* is overall mean expression level. τ_gender(i)_ is a fixed effect corresponding to gender for subject *i,* where *gender(⋅)* is a function mapping individual *i* to it's gender ∈ {male, female}. η_caseControl(i)_ is a fixed effect representing case/control status of subject *i,* where *caseControl(⋅)* is a function mapping individual *i* to it's case/control status for metabolic syndrome ∈ {case, control}, γ is a fixed effect incorporating the effect of age, α_batch(i,j)_ is a fixed effect included to accommodate an experimental batch effect (96-well plate format), where the function *batch(⋅)* is a function mapping each sample to a batch (plate) ∈ {1,2,3}. The κ_i_ are independently Gaussian distributed with mean zero and variance σ^2^_κ,_ for subject *i* representing inter individual variability. ε_ij_ is the residual error term incorporating technical (aliquot) variability, assumed to be independently Gaussian distributed with mean zero and variance σ^2^_ε_. The model was fitted in R, using the lme4 package[57] and the *lmer()* function, using maximum-likelihood. A likelihood ratio test was applied to assess the significance of the case-control effect (*η*). The p-value of the case-control effect in each model was calculated using a likelihood ratio (LR) test with the D = −2*log(LR) as the test statistic, which can be approximated by a Chi-square distribution with one degree of freedom. The p-values were adjusted for multiple testing by FDR correction [58]. In the replication study the batch effect was omitted from the model due to change of Illumina platform from 96-array format to 12-array format with no apparent batch differences present. Results were considered to be replicating if the fixed effect of interest (*η*) had a p-value<0.05 and consistent direction of the effect.

#### miRNA-mRNA analysis

The TargetScan database [37] was used to provide mRNA target predictions for each miRNA in the miRNA-mRNA analysis, here using predicted and conserved targets. TargetScan prediction was accessed through the R-package *targetscan.Hs.eg.db* (version 0.2.0). 248 of the miRNAs we have profiled have predicted conserved targets, for each one of these we assessed if there was an association with their target mRNAs. We defined an overall association through a statistical gene set test using the mean-rank gene set enrichment method (MR-GSE) [36]. We started with modelling the association between each miRNA and the full set of mRNAs profiled using a linear fixed-effects model with one mRNA as response variable, and the miRNA together with gender, age, metabolic syndrome case/control status, and batch as covariates. The mRNAs were ranked by their miRNA-associated t-statistic, in this case we were performing an one-sided test to assess if there were evidence for anti-correlated enrichment (i.e. negative t-statistic). The null hypothesis in the MR-GSE test is that the set of predicted target mRNAs are randomly chosen from the full set (i.e. all mRNAs profiled), p-values were calculated as for the Wilcoxon two-sample rank test [36]. The MR-GSE test was applied using functionality provided by the *limma*-package for R. P-values were adjusted for multiple testing by FDR correction [58] across the 248 performed enrichment tests. The miRNA-mRNA analysis was based upon 50 subjects with both miRNA and mRNA data available in abdominal adipose tissue and 55 subjects in gluteal adipose tissue.

#### Functional annotations using KEGG and miRNA-mRNA associations

The KEGG database [39] was used to provide an overall functional annotation based on detected miRNA-mRNA associations in each tissue. MiRNAs found to have a significant association with their mRNA target set using the above outlined gene set enrichment test (FDR adjusted p-value<0.05) were included in this analysis. KEGG terms corresponding to all mRNA targets for these miRNAs were aggregated within each tissue type. To test for significant enrichment of individual KEGG terms, a Fisher exact test was applied, comparing against the overall universe of KEGG terms in the full set of mRNAs profiled. P-values were subsequently adjusted for multiple testing by FDR correction [58].

## Supporting Information

Figure S1 Comparison of effect size estimates for miRNAs with tissue differential expression. (A) Effect size estimates from the joint analyis (both metabolic syndrome case and control subject included) vs. control subjects only. (B) Effect size estimates from the joint analyis (both metabolic syndrome case and control subject included) vs. metabolic syndrome case subjects only.
(TIF)

Figure S2 Reproducibility of estimated tissue differential miRNA expression effects (*β*). Plot of primary study coefficients *vs.* replication study coefficients for all miRNAs that were found to be significantly differentially expressed in the primary study. The linear relationship between the coefficients indicate that there is a relatively high-degree of concordance between the two studies. Error-bars indicate the standard-error of β for each miRNA.
(TIF)

Figure S3 Reproducibility of estimated metabolic syndrome associated differential miRNA expression effects in abdominal adipose tissue. Plot of primary study coefficients *vs.* replication study coefficients for all miRNAs that were found to be significantly differentially expressed in the primary study. Error-bars indicate the standard-error of β for each miRNA.
(TIF)

Figure S4 Reproducibility of estimated metabolic syndrome associated differential miRNA expression effects in gluteal adipose tissue. Plot of primary study coefficients *vs.* replication study coefficients for all miRNAs that were found to be significantly differentially expressed in the primary study. Error-bars indicate the standard-error of β for each miRNA.
(TIF)

Figure S5 Distribution of p-values for miRNA eQTL models. B) Quantile-Quantile plot of p-values in abdominal fat tissue (genomic control analysis [59] parameter λ = 1.12) C) Histogram of p-values in gluteal adipose tissue. D) Quantile-Quantile plot of p-values in abdominal fat tissue (genomic control analysis [59] parameter λ = 1.09).
(TIF)

Figure S6 Comparison of significant miRNAs across analyses. (A) Common significant miRNAs between tissue differential expression analysis (tissue) and metabolic syndrome case-control (Case-Con.) association, target mRNA association (mRNA) and miRNA eQTLs (eQTL) in abdominal adipose tissue. (B) Common significant miRNAs between tissue differential expression analysis (tissue) and metabolic syndrome case-control (Case-Con.) association, target mRNA association (mRNA) and miRNA eQTLs (eQTL) in gluteal adipose tissue.
(TIF)

Figure S7 Histogram of correlations between pairs of (technical) duplicate array profiles in the primary study. Results indicate a high degree of concordance between technical duplicate measurements.
(TIF)

Table S1 Candidate set of tissue differentially expressed miRNAs from the primary study together with results from the confirmation study.
(DOC)

Table S2 miRNA associated with metabolic syndrome case-control status in abdominal adipose tissue.
(DOC)

Table S3 miRNA associated with metabolic syndrome case-control status in gluteal adipose tissue.
(DOC)

Table S4 Candidate set of miRNA eQTLs in abdominal adipose tissue from the primary study together with results from the confirmation study.
(DOC)

Table S5 Candidate set miRNA eQTLs in gluteal adipose tissue from the primary study together with results from the confirmation study.
(DOC)

Table S6 Genome-wide association analysis results of eQTL SNPs in relation to BMI and waist/hip ratio adjusted for BMI.
(DOC)

Table S7 miRNAs significantly associated with their mRNA targets in abdominal adipose tissue.
(DOC)

Table S8 miRNAs significantly associated with their mRNA targets in gluteal adipose tissue.
(DOC)

Table S9 Significant KEGG terms from miRNA-mRNA association analysis in abdominal adipose tissue.
(DOC)

Table S10 Significant KEGG terms from miRNA-mRNA association analysis in gluteal adipose tissue.
(DOC)

Text S1 List of MolPAGE consortium partners.
(DOC)

Text S2 List of GIANT consortium partners.
(DOC)
