# Supplementary figures and images for: MicroRNA Expression in Abdominal and Gluteal Adipose Tissue Is Associated with mRNA Expression Levels and Partly Genetically Driven
Source: PLoS One. 2011 Nov 15;6(11):e27338. doi: 10.1371/journal.pone.0027338 (PMC3216936; doi:10.1371/journal.pone.0027338)

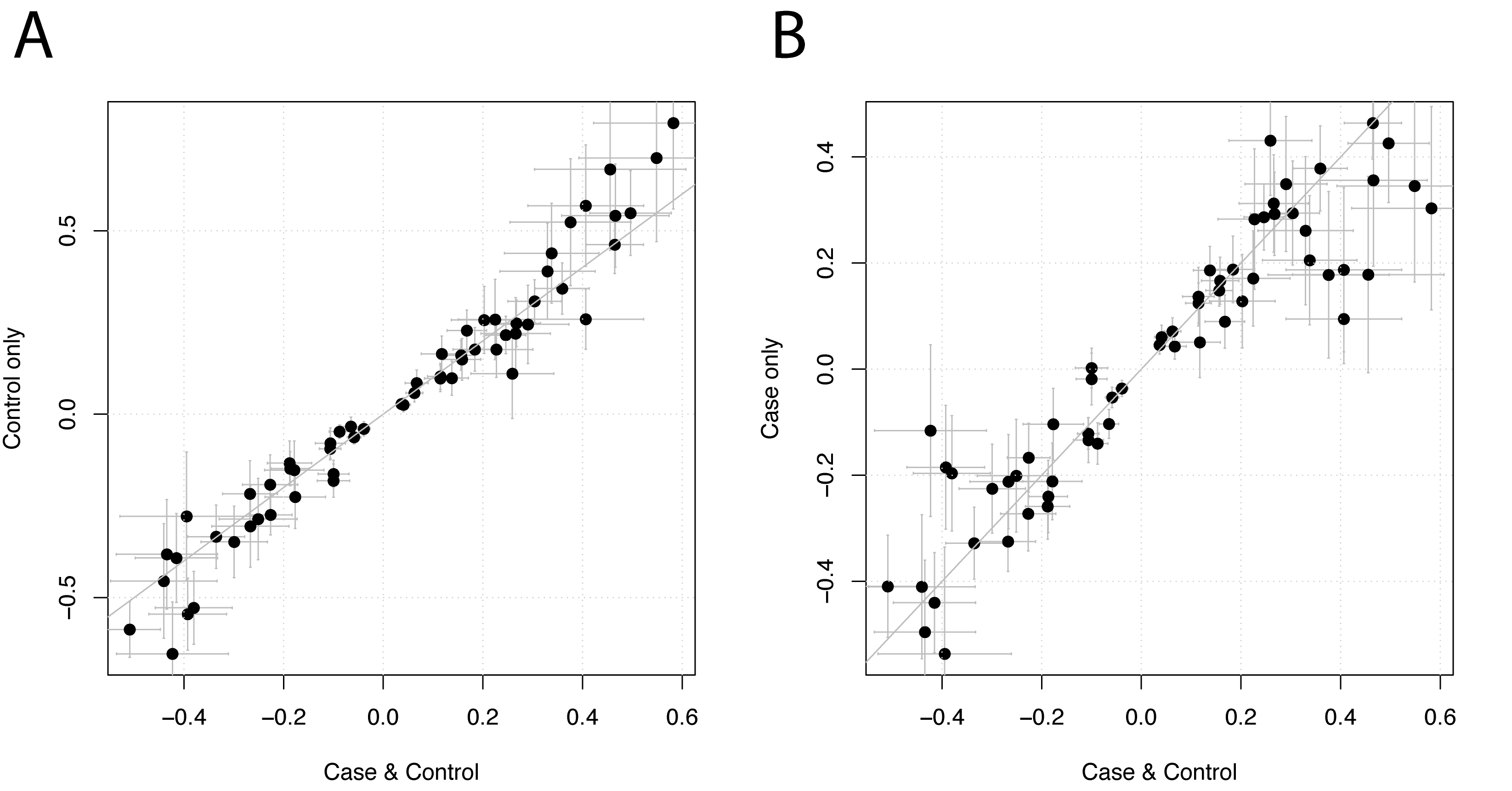

Supplement: Figure S1 — Comparison of effect size estimates for miRNAs with tissue differential expression. (A) Effect size estimates from the joint analyis (both metabolic syndrome case and control subject included) vs. control subjects only. (B) Effect size estimates from the joint analyis (both metabolic syndrome case and control subject included) vs. metabolic syndrome case subjects only. (TIF) [file pone.0027338.s001.tif]

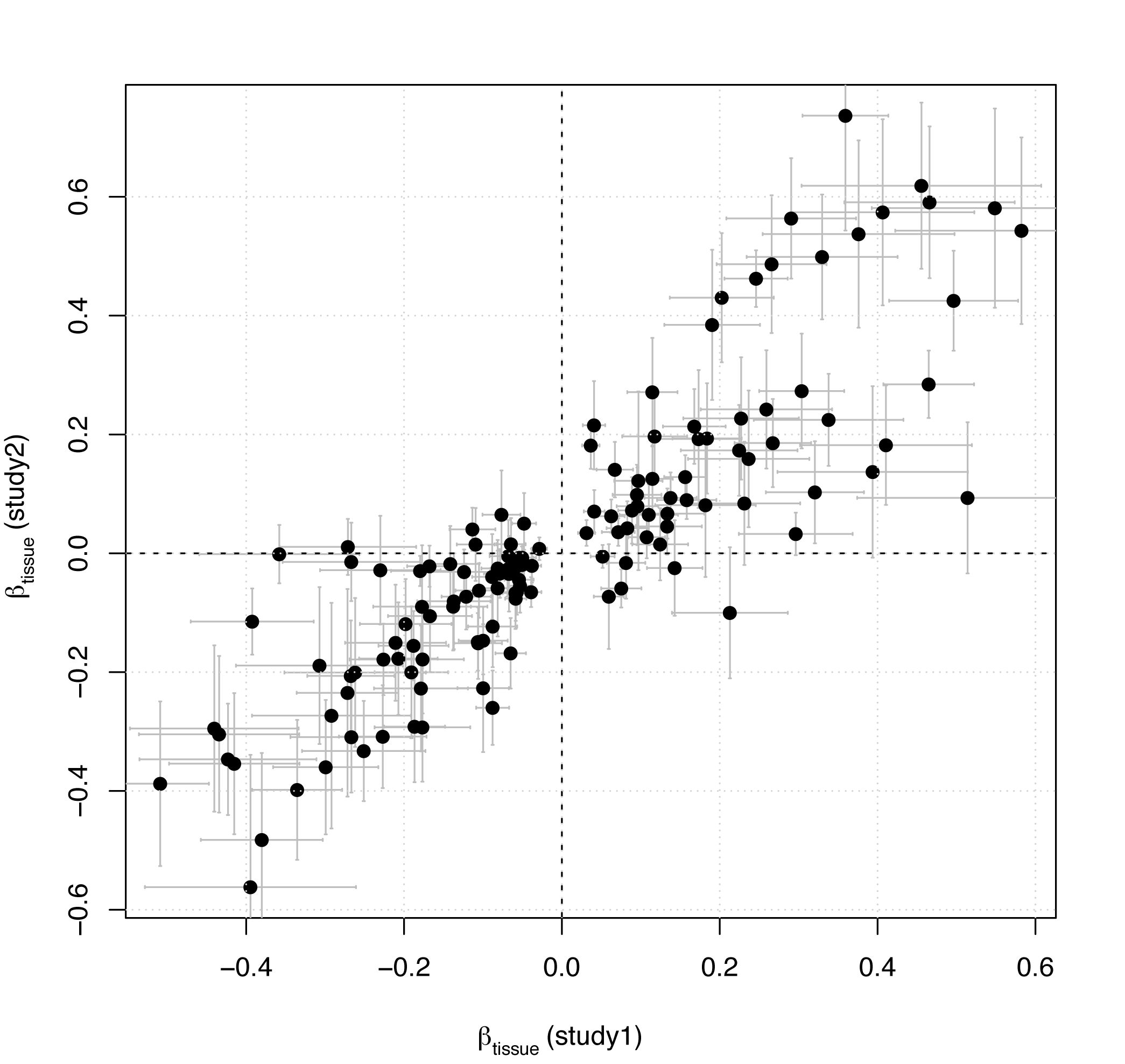

Supplement: Figure S2 — Reproducibility of estimated tissue differential miRNA expression effects (β). Plot of primary study coefficients vs. replication study coefficients for all miRNAs that were found to be significantly differentially expressed in the primary study. The linear relationship between the coefficients indicate that there is a relatively high-degree of concordance between the two studies. Error-bars indicate the standard-error of β for each miRNA. (TIF) [file pone.0027338.s002.tif]

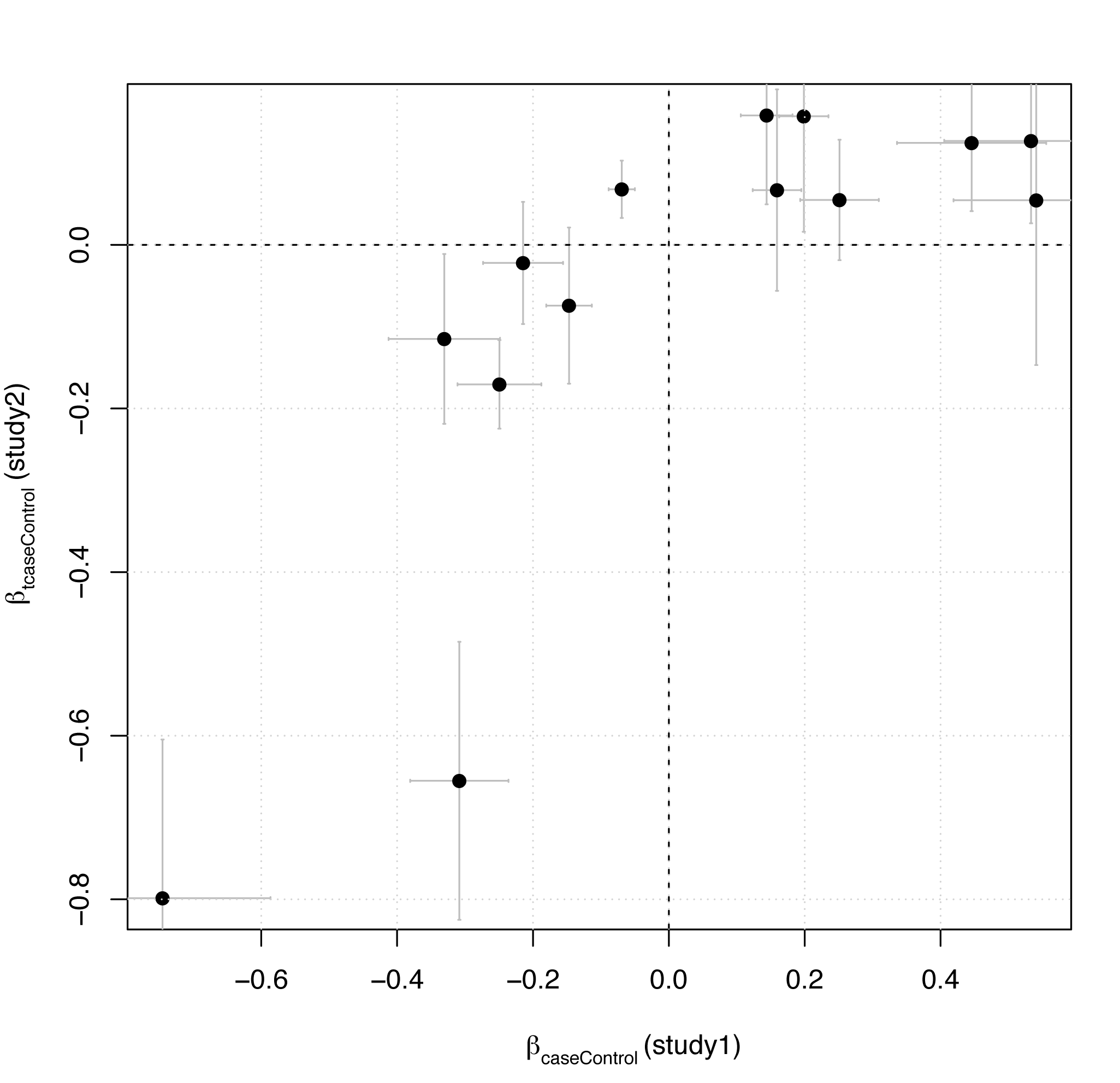

Supplement: Figure S3 — Reproducibility of estimated metabolic syndrome associated differential miRNA expression effects in abdominal adipose tissue. Plot of primary study coefficients vs. replication study coefficients for all miRNAs that were found to be significantly differentially expressed in the primary study. Error-bars indicate the standard-error of β for each miRNA. (TIF) [file pone.0027338.s003.tif]

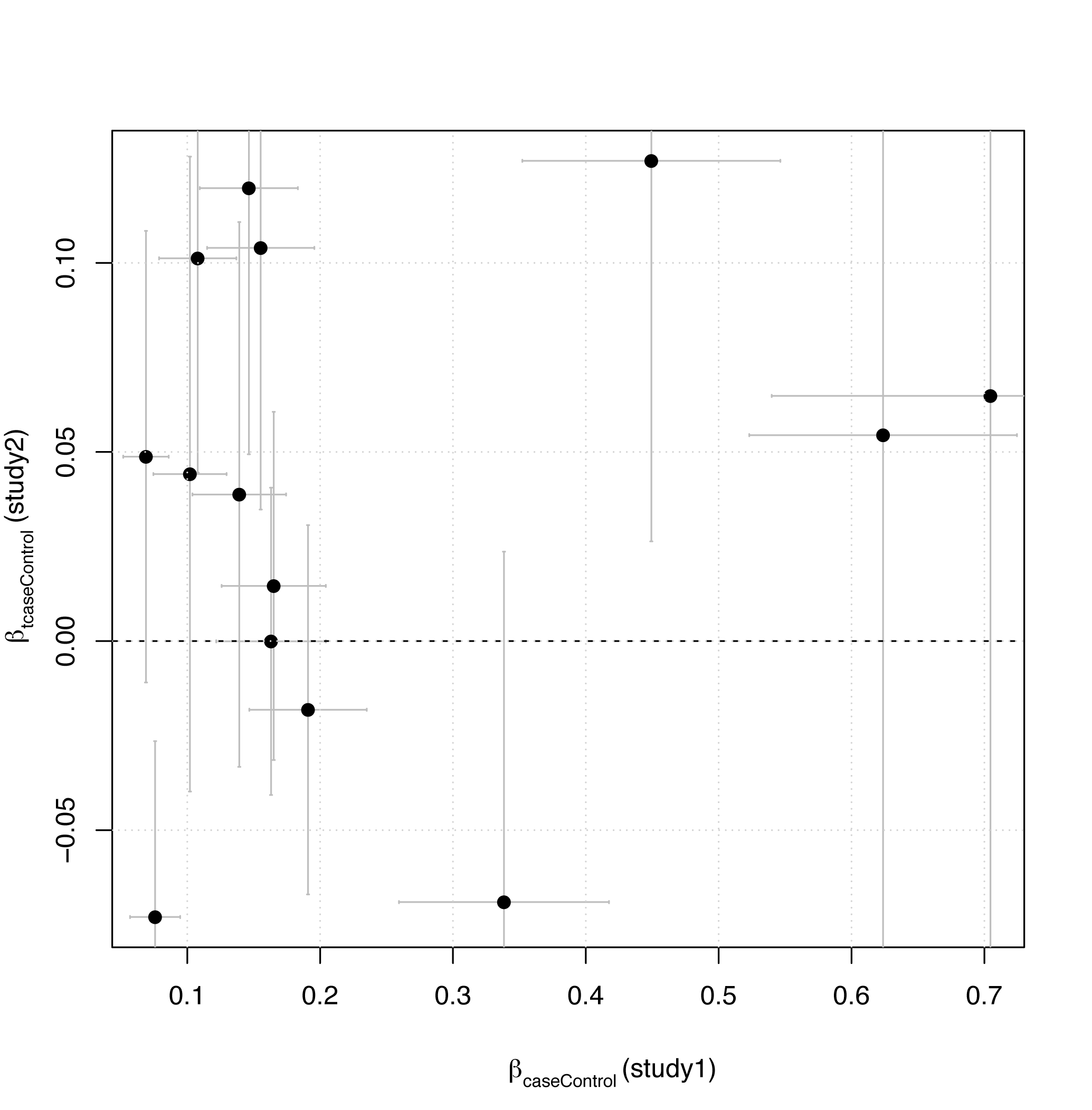

Supplement: Figure S4 — Reproducibility of estimated metabolic syndrome associated differential miRNA expression effects in gluteal adipose tissue. Plot of primary study coefficients vs. replication study coefficients for all miRNAs that were found to be significantly differentially expressed in the primary study. Error-bars indicate the standard-error of β for each miRNA. (TIF) [file pone.0027338.s004.tif]

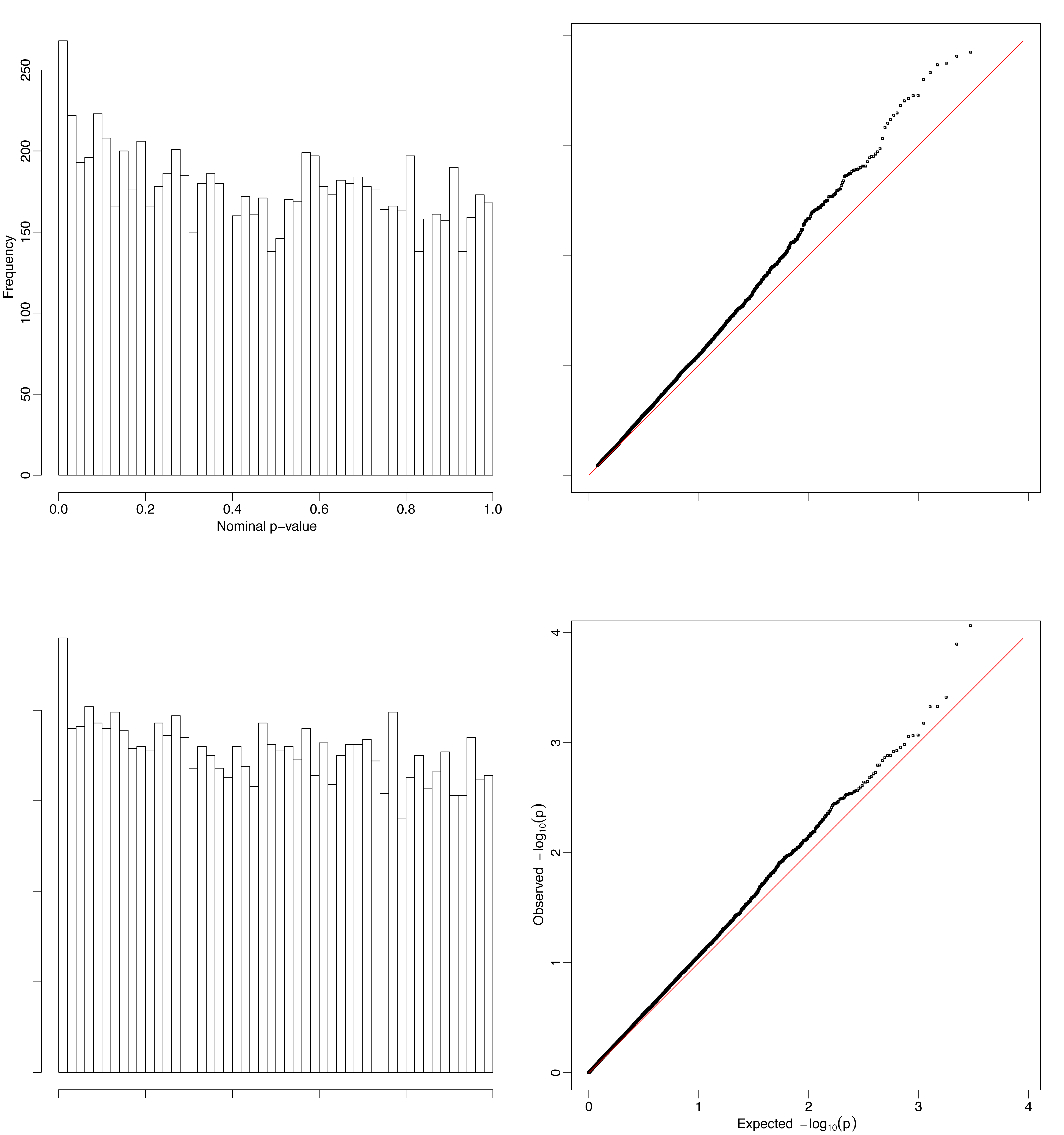

Supplement: Figure S5 — Distribution of p-values for miRNA eQTL models. B) Quantile-Quantile plot of p-values in abdominal fat tissue (genomic control analysis [59] parameter λ = 1.12) C) Histogram of p-values in gluteal adipose tissue. D) Quantile-Quantile plot of p-values in abdominal fat tissue (genomic control analysis [59] parameter λ = 1.09). (TIF) [file pone.0027338.s005.tif]

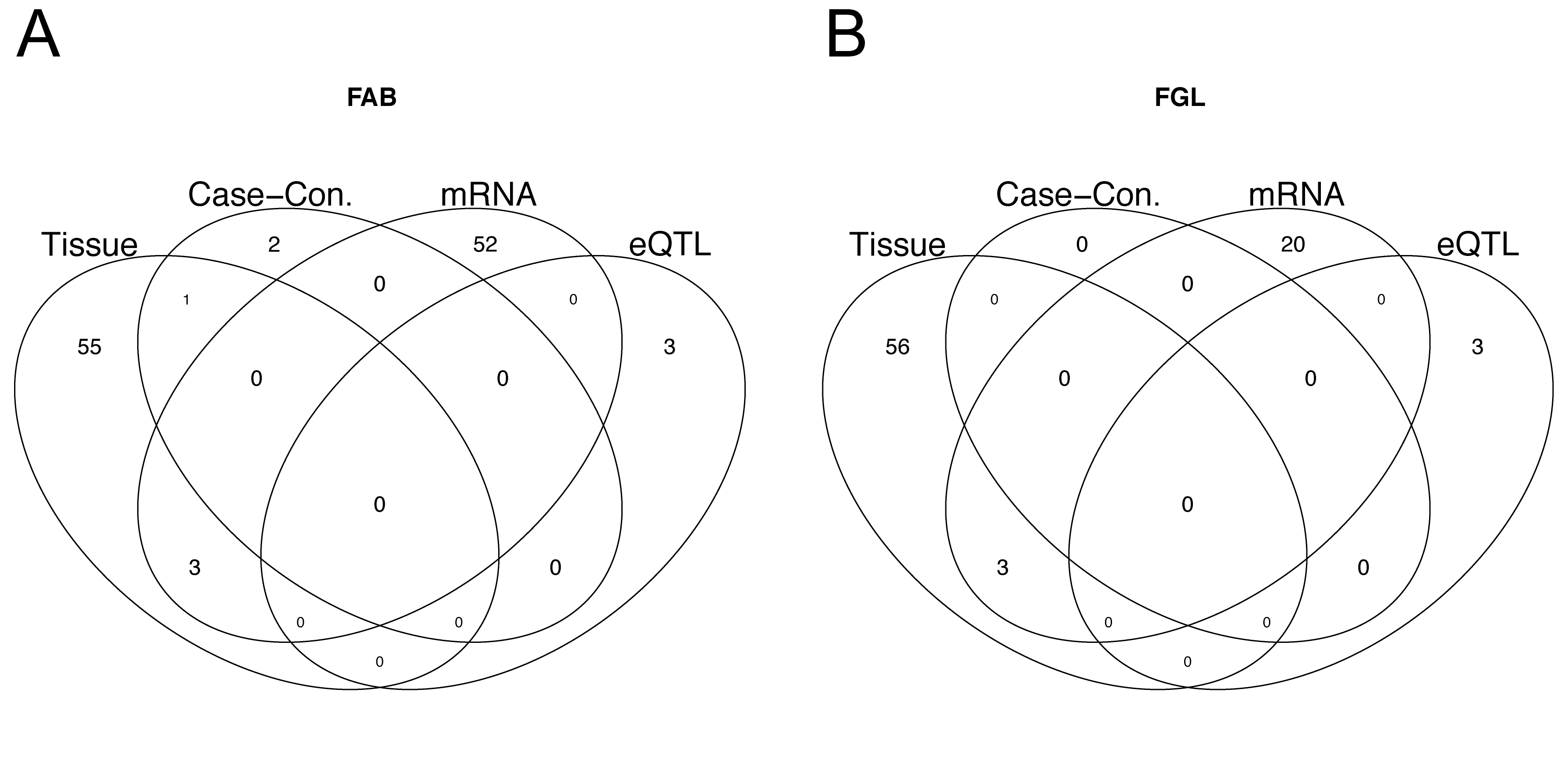

Supplement: Figure S6 — Comparison of significant miRNAs across analyses. (A) Common significant miRNAs between tissue differential expression analysis (tissue) and metabolic syndrome case-control (Case-Con.) association, target mRNA association (mRNA) and miRNA eQTLs (eQTL) in abdominal adipose tissue. (B) Common significant miRNAs between tissue differential expression analysis (tissue) and metabolic syndrome case-control (Case-Con.) association, target mRNA association (mRNA) and miRNA eQTLs (eQTL) in gluteal adipose tissue. (TIF) [file pone.0027338.s006.tif]

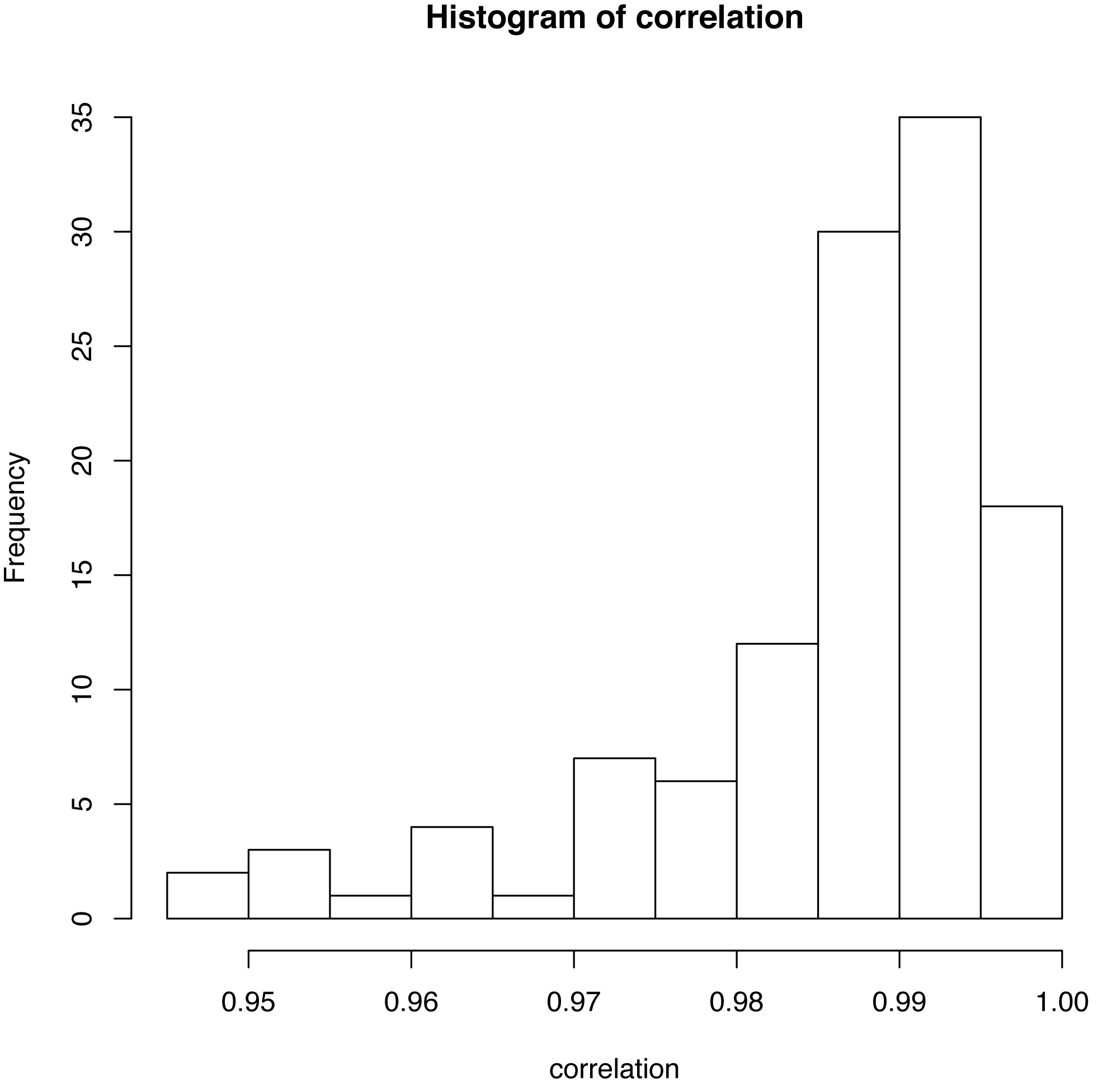

Supplement: Figure S7 — Histogram of correlations between pairs of (technical) duplicate array profiles in the primary study. Results indicate a high degree of concordance between technical duplicate measurements. (TIF) [file pone.0027338.s007.tif]
