# Supplementary material for: MicroRNA Expression in Abdominal and Gluteal Adipose Tissue Is Associated with mRNA Expression Levels and Partly Genetically Driven
Source: PLoS One. 2011 Nov 15;6(11):e27338. doi: 10.1371/journal.pone.0027338 (PMC3216936; doi:10.1371/journal.pone.0027338)
Supplement: Table S1 — Candidate set of tissue differentially expressed miRNAs from the primary study together with results from the confirmation study. (DOC) [file pone.0027338.s008.doc]

**Table S1.** Candidate set of tissue differentially expressed miRNAs from the primary study together with results from confirmation study.

|  |  | **Primary Study** | | | | **Replication study** | | |  |
| --- | --- | --- | --- | --- | --- | --- | --- | --- | --- |
| **miRNA**^a^ | **Avr.expr**^b^ | **β_tissue_** ^c^ | **s.e. (β_tissue_)**^d^ | **Nominal p-value**^e^ | **FDR adjusted p-value**^f^ | **β_tissue_ (study 2)**^g^ | **s.e. (β_tissue_) (study 2)**^h^ | **Nominal p-value (study 2)**^i^ | **Ref**^j^ |
| hsa-miR-196b | 12.438 | 0.465 | 0.057 | 5.12E-13 | 5.87E-10 | 0.284 | 0.057 | 1.39E-05 |  |
| hsa-miR-296-5p | 8.611 | -0.509 | 0.062 | 2.15E-11 | 1.23E-08 | -0.388 | 0.139 | 7.30E-03 |  |
| hsa-miR-1268 | 7.901 | 0.359 | 0.054 | 9.02E-10 | 3.44E-07 | 0.737 | 0.193 | 3.66E-04 |  |
| hsa-miR-196a | 14.009 | 0.246 | 0.04 | 2.12E-08 | 6.07E-06 | 0.462 | 0.048 | 2.92E-12 |  |
| hsa-miR-708 | 11.72 | 0.496 | 0.082 | 5.26E-08 | 1.21E-05 | 0.425 | 0.084 | 7.57E-06 |  |
| hsa-miR-423-3p | 13.348 | -0.168 | 0.028 | 8.48E-08 | 1.59E-05 | -0.022 | 0.035 | 5.31E-01 |  |
| HS_147 | 7.995 | 0.304 | 0.054 | 9.72E-08 | 1.59E-05 | 0.273 | 0.097 | 6.91E-03 |  |
| hsa-miR-27b | 13.808 | 0.156 | 0.027 | 1.26E-07 | 1.81E-05 | 0.128 | 0.037 | 1.02E-03 | [21] |
| HS_5.1 | 8.161 | -0.335 | 0.057 | 1.94E-07 | 2.48E-05 | -0.398 | 0.118 | 1.36E-03 |  |
| hsa-miR-1228* | 13.423 | 0.267 | 0.049 | 6.72E-07 | 7.71E-05 | 0.186 | 0.074 | 1.58E-02 |  |
| hsa-miR-326 | 12.189 | -0.392 | 0.078 | 1.57E-06 | 1.63E-04 | -0.115 | 0.056 | 4.86E-02 | [20] |
| hsa-miR-1288 | 7.674 | 0.089 | 0.018 | 1.73E-06 | 1.65E-04 | 0.072 | 0.056 | 2.05E-01 |  |
| hsa-miR-181a-2* | 11.173 | -0.226 | 0.043 | 1.92E-06 | 1.69E-04 | -0.179 | 0.06 | 3.78E-03 |  |
| hsa-miR-483-5p | 8.564 | 0.321 | 0.062 | 2.60E-06 | 2.13E-04 | 0.103 | 0.086 | 2.36E-01 |  |
| solexa-1460-671 | 9.35 | -0.415 | 0.083 | 3.20E-06 | 2.44E-04 | -0.354 | 0.119 | 4.91E-03 |  |
| hsa-miR-484 | 14.106 | -0.106 | 0.022 | 5.45E-06 | 3.91E-04 | -0.149 | 0.048 | 3.54E-03 |  |
| hsa-miR-331-3p | 13.519 | -0.18 | 0.037 | 6.77E-06 | 4.57E-04 | -0.03 | 0.025 | 2.44E-01 | [31] |
| hsa-miR-504 | 9.937 | -0.38 | 0.077 | 7.33E-06 | 4.66E-04 | -0.482 | 0.146 | 1.74E-03 |  |
| hsa-miR-636 | 7.939 | -0.267 | 0.055 | 8.26E-06 | 4.98E-04 | -0.206 | 0.093 | 3.17E-02 |  |
| hsa-miR-128a | 7.867 | -0.187 | 0.039 | 9.62E-06 | 5.51E-04 | -0.292 | 0.093 | 3.06E-03 |  |
| hsa-miR-887 | 7.833 | -0.067 | 0.014 | 1.28E-05 | 7.00E-04 | -0.034 | 0.064 | 5.89E-01 |  |
| hsa-miR-148b* | 8.141 | 0.108 | 0.023 | 1.61E-05 | 8.16E-04 | 0.027 | 0.036 | 4.45E-01 |  |
| hsa-miR-641 | 8.385 | -0.109 | 0.024 | 1.64E-05 | 8.16E-04 | 0.015 | 0.061 | 1.00E+00 |  |
| HS_305_b | 9.769 | -0.299 | 0.067 | 3.05E-05 | 1.46E-03 | -0.36 | 0.113 | 1.96E-03 |  |
| hsa-miR-187 | 7.983 | 0.115 | 0.026 | 3.31E-05 | 1.52E-03 | 0.126 | 0.056 | 2.97E-02 |  |
| hsa-miR-204 | 10.523 | 0.466 | 0.108 | 4.71E-05 | 2.07E-03 | 0.591 | 0.128 | 3.55E-05 | [22,23] |
| HS_3 | 7.137 | -0.058 | 0.013 | 5.34E-05 | 2.27E-03 | -0.076 | 0.038 | 4.45E-02 |  |
| hsa-miR-27b* | 9.821 | 0.184 | 0.044 | 6.36E-05 | 2.53E-03 | 0.193 | 0.093 | 4.03E-02 |  |
| hsa-miR-18a* | 11.227 | -0.434 | 0.102 | 6.40E-05 | 2.53E-03 | -0.305 | 0.132 | 2.55E-02 |  |
| hsa-miR-28-5p | 13.376 | 0.158 | 0.038 | 7.48E-05 | 2.76E-03 | 0.09 | 0.032 | 7.31E-03 | [33] |
| hsa-let-7i* | 7.624 | -0.272 | 0.064 | 7.75E-05 | 2.76E-03 | -0.235 | 0.175 | 1.83E-01 |  |
| hsa-miR-548d-3p | 8.366 | -0.188 | 0.044 | 7.78E-05 | 2.76E-03 | -0.156 | 0.058 | 9.51E-03 |  |
| hsa-miR-128b | 7.413 | -0.088 | 0.021 | 7.96E-05 | 2.76E-03 | -0.26 | 0.063 | 1.43E-04 |  |
| hsa-miR-138-1* | 8.09 | 0.296 | 0.072 | 8.72E-05 | 2.92E-03 | 0.033 | 0.036 | 3.63E-01 |  |
| hsa-miR-146a | 13.761 | 0.168 | 0.04 | 8.91E-05 | 2.92E-03 | 0.213 | 0.063 | 1.41E-03 |  |
| solexa-9578-86 | 9.649 | 0.138 | 0.033 | 1.03E-04 | 3.22E-03 | 0.093 | 0.043 | 3.25E-02 |  |
| hsa-miR-454* | 10.11 | -0.44 | 0.107 | 1.07E-04 | 3.22E-03 | -0.295 | 0.14 | 3.97E-02 |  |
| hsa-miR-1227 | 9.359 | -0.207 | 0.05 | 1.07E-04 | 3.22E-03 | -0.177 | 0.095 | 6.39E-02 |  |
| HS_142.1 | 9.861 | -0.227 | 0.056 | 1.22E-04 | 3.58E-03 | -0.309 | 0.087 | 7.91E-04 |  |
| hsa-miR-335* | 12.6 | -0.191 | 0.047 | 1.27E-04 | 3.64E-03 | -0.2 | 0.111 | 7.64E-02 | [31] |
| hsa-miR-491-3p | 9.082 | -0.113 | 0.029 | 1.48E-04 | 4.06E-03 | 0.04 | 0.036 | 1.00E+00 |  |
| hsa-miR-32* | 8 | 0.143 | 0.035 | 1.49E-04 | 4.06E-03 | -0.025 | 0.08 | 1.00E+00 |  |
| hsa-miR-324-3p | 13.426 | -0.121 | 0.031 | 1.66E-04 | 4.42E-03 | -0.073 | 0.055 | 1.98E-01 |  |
| solexa-7297-115 | 7.644 | -0.081 | 0.021 | 1.79E-04 | 4.67E-03 | -0.059 | 0.081 | 4.76E-01 |  |
| HS_303_a | 10.069 | -0.141 | 0.037 | 1.90E-04 | 4.84E-03 | -0.018 | 0.064 | 7.80E-01 |  |
| hsa-miR-609 | 8.896 | -0.081 | 0.021 | 2.40E-04 | 5.89E-03 | -0.025 | 0.03 | 3.98E-01 |  |
| hsa-miR-483-3p | 12.605 | 0.411 | 0.109 | 2.41E-04 | 5.89E-03 | 0.182 | 0.101 | 7.45E-02 |  |
| HS_122.1 | 8.124 | -0.058 | 0.015 | 2.54E-04 | 6.05E-03 | -0.023 | 0.03 | 4.43E-01 |  |
| hsa-miR-545* | 7.924 | -0.078 | 0.02 | 2.66E-04 | 6.22E-03 | -0.034 | 0.041 | 4.10E-01 |  |
| hsa-miR-511 | 11.555 | 0.266 | 0.07 | 2.74E-04 | 6.28E-03 | 0.487 | 0.116 | 1.40E-04 |  |
| hsa-miR-215 | 10.064 | -0.423 | 0.112 | 3.52E-04 | 7.92E-03 | -0.347 | 0.094 | 5.92E-04 |  |
| hsa-miR-1273 | 9.332 | 0.514 | 0.14 | 4.08E-04 | 8.99E-03 | 0.093 | 0.127 | 4.64E-01 |  |
| HS_215 | 7.535 | 0.063 | 0.017 | 4.76E-04 | 1.03E-02 | 0.063 | 0.028 | 3.01E-02 |  |
| hsa-miR-518e*,hsa-miR-519a*,hsa-miR-519b-5p,hsa-miR-519c-5p,hsa-miR-522*,hsa-miR-523* | 10.159 | 0.582 | 0.16 | 4.92E-04 | 1.04E-02 | 0.543 | 0.157 | 1.19E-03 |  |
| hsa-miR-361-5p | 13.281 | 0.096 | 0.026 | 5.00E-04 | 1.04E-02 | 0.079 | 0.065 | 2.31E-01 |  |
| hsa-miR-148a* | 7.619 | -0.059 | 0.016 | 5.45E-04 | 1.11E-02 | -0.067 | 0.06 | 2.73E-01 |  |
| hsa-miR-489 | 7.372 | 0.115 | 0.032 | 5.52E-04 | 1.11E-02 | 0.271 | 0.091 | 4.93E-03 |  |
| hsa-miR-1323 | 10.131 | 0.338 | 0.095 | 6.27E-04 | 1.24E-02 | 0.225 | 0.077 | 5.87E-03 |  |
| hsa-miR-649 | 7.474 | -0.048 | 0.014 | 6.74E-04 | 1.31E-02 | -0.018 | 0.024 | 4.56E-01 |  |
| hsa-miR-146b-5p | 12.493 | 0.291 | 0.082 | 7.18E-04 | 1.35E-02 | 0.564 | 0.101 | 1.28E-06 |  |
| hsa-miR-516b | 8.714 | 0.549 | 0.156 | 7.23E-04 | 1.35E-02 | 0.581 | 0.168 | 1.16E-03 |  |
| hsa-miR-148a | 13.859 | -0.105 | 0.03 | 7.31E-04 | 1.35E-02 | -0.063 | 0.046 | 1.82E-01 |  |
| hsa-miR-518f | 8.326 | 0.406 | 0.116 | 7.46E-04 | 1.36E-02 | 0.574 | 0.157 | 6.30E-04 |  |
| hsa-miR-28-3p | 12.765 | 0.124 | 0.035 | 7.96E-04 | 1.42E-02 | 0.015 | 0.061 | 8.04E-01 |  |
| hsa-miR-363* | 7.257 | -0.069 | 0.019 | 8.05E-04 | 1.42E-02 | -0.027 | 0.045 | 5.47E-01 |  |
| hsa-miR-1208 | 8.305 | -0.065 | 0.019 | 8.46E-04 | 1.44E-02 | 0.015 | 0.044 | 1.00E+00 |  |
| hsa-miR-554 | 9.08 | -0.106 | 0.03 | 8.54E-04 | 1.44E-02 | -0.151 | 0.06 | 1.51E-02 |  |
| hsa-miR-10b | 12.814 | -0.358 | 0.101 | 8.65E-04 | 1.44E-02 | -0.001 | 0.049 | 9.77E-01 | [33] |
| hsa-miR-1229 | 9.139 | -0.267 | 0.077 | 8.66E-04 | 1.44E-02 | -0.309 | 0.093 | 1.60E-03 |  |
| solexa-539-2056 | 9.467 | 0.33 | 0.096 | 9.08E-04 | 1.49E-02 | 0.499 | 0.105 | 2.09E-05 |  |
| hsa-miR-211 | 7.948 | -0.088 | 0.025 | 9.47E-04 | 1.53E-02 | -0.04 | 0.072 | 5.82E-01 | [22,23] |
| hsa-miR-297 | 7.902 | -0.05 | 0.015 | 9.99E-04 | 1.58E-02 | -0.019 | 0.031 | 5.31E-01 |  |
| hsa-miR-128 | 11.844 | -0.198 | 0.058 | 1.03E-03 | 1.58E-02 | -0.119 | 0.076 | 1.20E-01 |  |
| HS_135 | 7.265 | 0.037 | 0.011 | 1.03E-03 | 1.58E-02 | 0.182 | 0.039 | 4.46E-05 |  |
| hsa-miR-27a | 14.083 | 0.071 | 0.021 | 1.03E-03 | 1.58E-02 | 0.036 | 0.023 | 1.29E-01 | [24,25,31] |
| HS_208 | 7.552 | -0.053 | 0.015 | 1.09E-03 | 1.64E-02 | -0.055 | 0.045 | 2.34E-01 |  |
| hsa-miR-143 | 14.221 | 0.052 | 0.015 | 1.17E-03 | 1.72E-02 | -0.005 | 0.019 | 1.00E+00 | [26,27,29,33] |
| hsa-miR-668 | 8.203 | -0.176 | 0.052 | 1.17E-03 | 1.72E-02 | -0.178 | 0.091 | 5.52E-02 |  |
| hsa-miR-486-5p | 14.61 | -0.065 | 0.019 | 1.32E-03 | 1.92E-02 | -0.168 | 0.059 | 7.03E-03 |  |
| hsa-miR-130b* | 10.003 | -0.211 | 0.064 | 1.36E-03 | 1.95E-02 | -0.151 | 0.098 | 1.29E-01 |  |
| HS_179 | 7.407 | -0.038 | 0.012 | 1.45E-03 | 2.05E-02 | -0.021 | 0.029 | 4.65E-01 |  |
| hsa-miR-1285 | 9.94 | 0.394 | 0.121 | 1.50E-03 | 2.10E-02 | 0.137 | 0.144 | 3.45E-01 |  |
| HS_54 | 8.628 | 0.231 | 0.071 | 1.60E-03 | 2.21E-02 | 0.084 | 0.104 | 4.19E-01 |  |
| hsa-miR-376b | 8.907 | 0.081 | 0.024 | 1.63E-03 | 2.22E-02 | -0.016 | 0.06 | 1.00E+00 |  |
| hsa-miR-26b* | 10.613 | -0.138 | 0.043 | 1.90E-03 | 2.53E-02 | -0.09 | 0.074 | 2.26E-01 |  |
| hsa-miR-1287 | 8.856 | 0.173 | 0.054 | 1.91E-03 | 2.53E-02 | 0.192 | 0.116 | 1.01E-01 |  |
| hsa-miR-598 | 10.23 | -0.251 | 0.078 | 1.92E-03 | 2.53E-02 | -0.333 | 0.084 | 1.81E-04 |  |
| hsa-miR-23b | 14.449 | 0.041 | 0.013 | 2.06E-03 | 2.63E-02 | 0.07 | 0.036 | 5.81E-02 |  |
| solexa-9655-85 | 10.019 | -0.1 | 0.031 | 2.06E-03 | 2.63E-02 | -0.147 | 0.057 | 1.29E-02 |  |
| HS_268 | 8.909 | -0.077 | 0.024 | 2.24E-03 | 2.79E-02 | 0.065 | 0.074 | 1.00E+00 |  |
| hsa-miR-373 | 7.203 | 0.097 | 0.031 | 2.24E-03 | 2.79E-02 | 0.122 | 0.15 | 4.19E-01 |  |
| hsa-miR-384 | 7.641 | -0.048 | 0.015 | 2.37E-03 | 2.93E-02 | 0.05 | 0.051 | 1.00E+00 |  |
| hsa-miR-15a* | 7.229 | 0.059 | 0.019 | 2.49E-03 | 3.03E-02 | -0.073 | 0.088 | 1.00E+00 |  |
| hsa-miR-520c-3p,hsa-miR-520f | 7.558 | 0.203 | 0.066 | 2.53E-03 | 3.03E-02 | 0.43 | 0.109 | 2.57E-04 |  |
| hsa-miR-885-3p | 7.67 | 0.083 | 0.026 | 2.55E-03 | 3.03E-02 | 0.042 | 0.045 | 3.51E-01 |  |
| HS_7 | 7.793 | 0.095 | 0.031 | 2.57E-03 | 3.03E-02 | 0.098 | 0.051 | 5.62E-02 |  |
| hsa-miR-339-5p | 10.811 | -0.267 | 0.087 | 2.64E-03 | 3.07E-02 | -0.014 | 0.066 | 8.27E-01 |  |
| hsa-miR-92b | 12.345 | -0.167 | 0.053 | 2.65E-03 | 3.07E-02 | -0.106 | 0.094 | 2.64E-01 |  |
| hsa-miR-519d | 8.276 | 0.376 | 0.122 | 2.71E-03 | 3.11E-02 | 0.537 | 0.158 | 1.34E-03 | [30] |
| hsa-miR-525-3p | 9.22 | 0.133 | 0.044 | 2.78E-03 | 3.14E-02 | 0.045 | 0.048 | 3.48E-01 |  |
| hsa-miR-675 | 10.236 | 0.237 | 0.077 | 2.80E-03 | 3.14E-02 | 0.159 | 0.115 | 1.70E-01 |  |
| hsa-miR-10a | 12.864 | -0.271 | 0.087 | 2.85E-03 | 3.17E-02 | 0.011 | 0.047 | 1.00E+00 | [33] |
| hsa-miR-155 | 12.893 | 0.227 | 0.073 | 2.96E-03 | 3.26E-02 | 0.227 | 0.103 | 3.42E-02 |  |
| solexa-3927-221 | 14.371 | -0.054 | 0.018 | 3.13E-03 | 3.42E-02 | -0.007 | 0.01 | 4.46E-01 |  |
| hsa-miR-7-2* | 7.277 | -0.1 | 0.033 | 3.19E-03 | 3.42E-02 | -0.227 | 0.108 | 3.96E-02 |  |
| hsa-miR-34a | 11.375 | 0.225 | 0.074 | 3.21E-03 | 3.42E-02 | 0.173 | 0.076 | 2.73E-02 | [23,33] |
| hsa-miR-628-5p | 13.015 | 0.259 | 0.083 | 3.22E-03 | 3.42E-02 | 0.242 | 0.1 | 1.94E-02 |  |
| hsa-miR-564 | 9.215 | 0.075 | 0.025 | 3.50E-03 | 3.68E-02 | -0.059 | 0.032 | 1.00E+00 |  |
| hsa-miR-520h,hsa-miR-520g | 8.922 | 0.455 | 0.152 | 3.58E-03 | 3.73E-02 | 0.619 | 0.14 | 5.95E-05 |  |
| hsa-miR-93* | 8.431 | -0.179 | 0.06 | 3.69E-03 | 3.81E-02 | -0.227 | 0.106 | 3.80E-02 |  |
| HS_199 | 8.226 | -0.068 | 0.023 | 3.75E-03 | 3.83E-02 | -0.026 | 0.04 | 5.08E-01 |  |
| solexa-3464-254 | 7.405 | -0.057 | 0.019 | 3.78E-03 | 3.83E-02 | -0.065 | 0.038 | 9.10E-02 |  |
| hsa-miR-184 | 7.024 | -0.039 | 0.013 | 3.89E-03 | 3.90E-02 | -0.065 | 0.025 | 1.24E-02 |  |
| hsa-miR-222 | 12.73 | 0.11 | 0.037 | 3.91E-03 | 3.90E-02 | 0.065 | 0.051 | 2.10E-01 | [23,25] |
| hsa-miR-365 | 13.197 | -0.23 | 0.076 | 4.07E-03 | 4.02E-02 | -0.028 | 0.091 | 7.56E-01 | [33] |
| hsa-miR-106b* | 10.462 | -0.177 | 0.061 | 4.20E-03 | 4.08E-02 | -0.293 | 0.092 | 2.31E-03 |  |
| hsa-miR-503 | 11.746 | -0.137 | 0.046 | 4.20E-03 | 4.08E-02 | -0.081 | 0.081 | 3.21E-01 | [33] |
| solexa-7111-119 | 7.167 | 0.031 | 0.011 | 4.26E-03 | 4.10E-02 | 0.034 | 0.022 | 1.19E-01 |  |
| hsa-miR-183 | 11.568 | -0.395 | 0.134 | 4.30E-03 | 4.10E-02 | -0.562 | 0.223 | 1.53E-02 |  |
| hsa-miR-199a-5p | 13.762 | -0.124 | 0.042 | 4.42E-03 | 4.19E-02 | -0.031 | 0.038 | 4.11E-01 | [31] |
| hsa-miR-7 | 12.55 | -0.262 | 0.09 | 4.52E-03 | 4.21E-02 | -0.201 | 0.125 | 1.15E-01 |  |
| hsa-miR-553 | 7.24 | -0.054 | 0.019 | 4.55E-03 | 4.21E-02 | -0.044 | 0.041 | 2.89E-01 |  |
| hsa-miR-552 | 7.209 | -0.029 | 0.01 | 4.56E-03 | 4.21E-02 | 0.008 | 0.019 | 1.00E+00 |  |
| hsa-miR-572 | 7.525 | 0.041 | 0.014 | 4.60E-03 | 4.21E-02 | 0.216 | 0.074 | 5.74E-03 |  |
| hsa-miR-532-5p | 11.463 | 0.134 | 0.047 | 4.73E-03 | 4.30E-02 | 0.067 | 0.042 | 1.19E-01 |  |
| hsa-miR-195 | 14.277 | 0.067 | 0.023 | 4.80E-03 | 4.33E-02 | 0.141 | 0.047 | 4.15E-03 | [33] |
| hsa-miR-520g | 7.217 | 0.118 | 0.041 | 4.90E-03 | 4.38E-02 | 0.197 | 0.076 | 1.12E-02 |  |
| hsa-miR-363 | 12.185 | -0.307 | 0.106 | 4.96E-03 | 4.38E-02 | -0.189 | 0.132 | 1.55E-01 |  |
| hsa-miR-15b* | 11.476 | -0.292 | 0.101 | 4.97E-03 | 4.38E-02 | -0.273 | 0.19 | 1.56E-01 |  |
| hsa-miR-605 | 7.417 | -0.05 | 0.018 | 5.41E-03 | 4.73E-02 | -0.007 | 0.023 | 7.48E-01 |  |
| hsa-let-7c* | 7.682 | -0.088 | 0.031 | 5.50E-03 | 4.77E-02 | -0.123 | 0.069 | 7.48E-02 |  |
| hsa-miR-125b-2* | 9.021 | -0.177 | 0.062 | 5.76E-03 | 4.95E-02 | -0.09 | 0.103 | 3.86E-01 |  |
| hsa-miR-190b | 10.575 | 0.213 | 0.073 | 5.83E-03 | 4.95E-02 | -0.1 | 0.11 | 1.00E+00 |  |
| hsa-miR-98 | 13.578 | 0.182 | 0.064 | 5.83E-03 | 4.95E-02 | 0.081 | 0.121 | 5.05E-01 | [33] |
| HS_280_a | 7.627 | -0.068 | 0.024 | 5.87E-03 | 4.95E-02 | -0.005 | 0.061 | 9.38E-01 |  |
| ^a^miRNA name**,** ^b^average miRNA expression in primary study**,** ^c^coefficient for the tissue effect in the primary study**,** ^d^standard error for the tissue coefficient in the primary study**,** ^e^p-value for the tissue effect in the primary study**,** ^f^FDR adjusted p-value for the tissue effect in the primary study**,** ^g^coefficient for the tissue effect in the replication study**,** ^h^standard error for the tissue coefficient in the replication study**, ^i^**p-value for the tissue effect in the replication study**,** ^j^References. | | | | | | | | | |
