# Supplementary material for: MicroRNA Expression in Abdominal and Gluteal Adipose Tissue Is Associated with mRNA Expression Levels and Partly Genetically Driven
Source: PLoS One. 2011 Nov 15;6(11):e27338. doi: 10.1371/journal.pone.0027338 (PMC3216936; doi:10.1371/journal.pone.0027338)
Supplement: Table S2 — miRNA associated with metabolic syndrome case-control status in abdominal adipose tissue. (DOC) [file pone.0027338.s009.doc]

**Table S2.** miRNA associated with metabolic syndrome case-control status in abdominal adipose tissue.

|  |  |  | **Primary study** | |  | **Replication study** | | |  |
| --- | --- | --- | --- | --- | --- | --- | --- | --- | --- |
| **miRNA**^a^ | **Average Expression**^b^ | **β**^c^ | **s.e.(β)**^d^ | **Nominal p-value**^e^ | **FDR adjusted p-value**^f^ | **β (study 2)**^g^ | **s.e.( β) (study 2)**^h^ | **Nominal p-value (study 2)**^i^ | **Ref**^j^ |
| hsa-miR-34a* | 7.588 | 0.199 | 0.036 | 6.95E-07 | 0.001 | 0.157 | 0.141 | 2.70E-01 |  |
| hsa-miR-1179 | 10.499 | -0.745 | 0.159 | 1.41E-05 | 0.008 | -0.799 | 0.194 | 1.85E-04 |  |
| hsa-miR-31* | 7.480 | 0.159 | 0.036 | 3.00E-05 | 0.009 | 0.067 | 0.123 | 5.87E-01 |  |
| hsa-miR-511 | 11.411 | 0.541 | 0.122 | 3.49E-05 | 0.009 | 0.054 | 0.201 | 7.87E-01 |  |
| hsa-miR-551b* | 7.293 | -0.147 | 0.034 | 4.18E-05 | 0.009 | -0.074 | 0.095 | 4.37E-01 |  |
| hsa-miR-532-5p | 11.399 | 0.251 | 0.058 | 4.61E-05 | 0.009 | 0.055 | 0.074 | 4.59E-01 |  |
| hsa-miR-7-2* | 7.325 | -0.308 | 0.072 | 6.34E-05 | 0.010 | -0.655 | 0.170 | 3.82E-04 |  |
| hsa-miR-34a | 11.249 | 0.533 | 0.128 | 8.88E-05 | 0.013 | 0.127 | 0.101 | 2.10E-01 | [23,33] |
| hsa-miR-652 | 12.915 | -0.249 | 0.062 | 1.36E-04 | 0.014 | -0.171 | 0.054 | 2.83E-03 |  |
| hsa-miR-198 | 8.037 | 0.446 | 0.110 | 1.37E-04 | 0.014 | 0.125 | 0.083 | 1.44E-01 |  |
| hsa-miR-1227 | 9.458 | -0.331 | 0.082 | 1.38E-04 | 0.014 | -0.115 | 0.104 | 2.72E-01 |  |
| hsa-miR-801 | 7.417 | 0.144 | 0.038 | 3.12E-04 | 0.030 | 0.158 | 0.109 | 1.48E-01 |  |
| hsa-miR-877* | 8.001 | -0.215 | 0.059 | 4.95E-04 | 0.044 | -0.022 | 0.075 | 7.67E-01 |  |
| HS_273 | 7.728 | -0.069 | 0.019 | 5.54E-04 | 0.045 | 0.068 | 0.035 | 1.00E+00 |  |
| ^a^miRNA name**,** ^b^average miRNA expression in primary study**,** ^c^coefficient for the tissue effect in the primary study**,** ^d^standard error for the tissue coefficient in the primary study**,** ^e^p-value for the tissue effect in the primary study**,** ^f^FDR adjusted p-value for the tissue effect in the primary study**,** ^g^coefficient for the tissue effect in the replication study**,** ^h^standard error for the tissue coefficient in the replication study**, ^i^**p-value for the tissue effect in the replication study**,** ^j^References. | | | | | | | | | |
