# Supplementary material for: MicroRNA Expression in Abdominal and Gluteal Adipose Tissue Is Associated with mRNA Expression Levels and Partly Genetically Driven
Source: PLoS One. 2011 Nov 15;6(11):e27338. doi: 10.1371/journal.pone.0027338 (PMC3216936; doi:10.1371/journal.pone.0027338)
Supplement: Table S3 — miRNA associated with metabolic syndrome case-control status in gluteal adipose tissue. (DOC) [file pone.0027338.s010.doc]

**Table S3.** miRNA associated with metabolic syndrome case-control status in gluteal adipose tissue.

|  |  | **Primary study** | | | | **replication study** | | |  |
| --- | --- | --- | --- | --- | --- | --- | --- | --- | --- |
| **miRNA**^a^ | **Average Expression**^b^ | **β**^c^ | **s.e.( β)**^d^ | **Nominal p-value**^e^ | **FDR adjusted p-value**^f^ | **β (study 2)**^g^ | **s.e.( β) (study 2)**^h^ | **Nominal p-value (study 2)**^i^ | **Ref**^j^ |
| hsa-miR-511 | 11.709 | 0.624 | 0.101 | 4.89E-08 | 0 | 0.054 | 0.201 | 7.87E-01 |  |
| hsa-miR-34a | 11.51 | 0.449 | 0.097 | 1.87E-05 | 0.011 | 0.127 | 0.101 | 2.10E-01 | [23,33] |
| hsa-miR-1204 | 9.178 | 0.191 | 0.044 | 5.21E-05 | 0.013 | -0.018 | 0.049 | 1.00E+00 |  |
| HS_94 | 9.546 | 0.338 | 0.079 | 5.53E-05 | 0.013 | -0.069 | 0.093 | 1.00E+00 |  |
| hsa-miR-146b-5p | 12.658 | 0.705 | 0.165 | 6.31E-05 | 0.013 | 0.065 | 0.177 | 7.14E-01 |  |
| hsa-miR-453 | 7.786 | 0.165 | 0.039 | 6.94E-05 | 0.013 | 0.015 | 0.046 | 7.52E-01 |  |
| hsa-miR-767-5p | 9.094 | 0.146 | 0.037 | 1.22E-04 | 0.02 | 0.12 | 0.07 | 9.47E-02 |  |
| hsa-miR-29a | 14.169 | 0.076 | 0.019 | 1.55E-04 | 0.02 | -0.073 | 0.047 | 1.00E+00 |  |
| HS_186 | 8.769 | 0.163 | 0.041 | 1.79E-04 | 0.02 | 0 | 0.041 | 1.00E+00 |  |
| HS_73.1 | 7.131 | 0.069 | 0.017 | 1.79E-04 | 0.02 | 0.049 | 0.06 | 4.15E-01 |  |
| HS_27 | 8.562 | 0.139 | 0.035 | 1.90E-04 | 0.02 | 0.039 | 0.072 | 5.91E-01 |  |
| hsa-miR-571 | 9.219 | 0.155 | 0.04 | 2.88E-04 | 0.027 | 0.104 | 0.069 | 1.38E-01 |  |
| hsa-miR-1184 | 8.986 | 0.102 | 0.028 | 3.86E-04 | 0.034 | 0.044 | 0.084 | 5.99E-01 |  |
| HS_100 | 14.066 | 0.108 | 0.029 | 4.92E-04 | 0.04 | 0.101 | 0.057 | 8.11E-02 |  |
| ^a^miRNA name**,** ^b^average miRNA expression in primary study**,** ^c^coefficient for the tissue effect in the primary study**,** ^d^standard error for the tissue coefficient in the primary study**,** ^e^p-value for the tissue effect in the primary study**,** ^f^FDR adjusted p-value for the tissue effect in the primary study**,** ^g^coefficient for the tissue effect in the replication study**,** ^h^standard error for the tissue coefficient in the replication study**, ^i^**p-value for the tissue effect in the replication study**,** ^j^References | | | | | | | | | |
