# Supplementary material for: MicroRNA Expression in Abdominal and Gluteal Adipose Tissue Is Associated with mRNA Expression Levels and Partly Genetically Driven
Source: PLoS One. 2011 Nov 15;6(11):e27338. doi: 10.1371/journal.pone.0027338 (PMC3216936; doi:10.1371/journal.pone.0027338)
Supplement: Table S4 — Candidate set of miRNA eQTLs in abdominal adipose tissue from the primary study together with results from the confirmation study. (DOC) [file pone.0027338.s011.doc]

**Table S4.** Candidate set of miRNA eQTLs in abdominal adipose tissue from the primary study together with results from confirmation study.

|  |  |  |  |  | **Primary study** | | | | **Replication study** | | |  |
| --- | --- | --- | --- | --- | --- | --- | --- | --- | --- | --- | --- | --- |
| **miRNA**^a^ | **SNP(rs)**^b^ | **Chr**^c^ | **SNP (Position)**^d^ | **Effect allele**^e^ | **β_snp_**^f^ | **std.error β_snp_**^g^ | **p-value**^h^ | **FDR adjusted p-value**^i^ | **β_snp_ (study2)**^j^ | **std.error β_snp_ (study2)**^k^ | **p-value (study2)**^l^ | **Ref**^m^ |
| hsa-miR-1255a | rs1476159 | 4 | 102500118 | T | 0.133 | 0.029 | 1.83E-05 | 0.081 | 0.164 | 0.063 | 1.39E-02 |  |
| hsa-miR-1255a | rs1822168 | 4 | 102512567 | C | 0.133 | 0.029 | 1.83E-05 | 0.081 | 0.161 | 0.060 | 9.91E-03 |  |
| hsa-miR-1255a | rs10516469 | 4 | 102480276 | T | 0.181 | 0.045 | 1.43E-04 | 0.276 | NA | NA | NA |  |
| hsa-miR-618 | rs10862209 | 12 | 79851086 | A | 0.268 | 0.067 | 1.55E-04 | 0.276 | NA | NA | NA |  |
| hsa-miR-34b | rs4938784 | 11 | 110918675 | T | -0.176 | 0.045 | 1.80E-04 | 0.276 | NA | NA | NA | [23] |
| hsa-miR-31 | rs991794 | 9 | 21541513 | C | -0.156 | 0.039 | 1.86E-04 | 0.276 | 0.053 | 0.116 | 1.00E+00 |  |
| hsa-miR-585 | rs1345592 | 5 | 168573568 | G | 0.195 | 0.050 | 2.18E-04 | 0.277 | 0.201 | 0.107 | 6.53E-02 |  |
| hsa-miR-1244 | rs838436 | 2 | 232291757 | G | -0.277 | 0.071 | 2.53E-04 | 0.282 | -0.057 | 0.098 | 5.66E-01 |  |
| hsa-miR-93* | rs12705070 | 7 | 99494863 | G | -0.268 | 0.071 | 3.53E-04 | 0.294 | -0.268 | 0.141 | 6.37E-02 | [33] |
| hsa-miR-93* | rs2070215 | 7 | 99534733 | C | -0.268 | 0.071 | 3.53E-04 | 0.294 | -0.268 | 0.141 | 6.37E-02 | [33] |
| hsa-miR-1307 | rs11191686 | 10 | 105177736 | A | 0.223 | 0.060 | 3.76E-04 | 0.294 | 0.119 | 0.109 | 2.79E-01 |  |
| hsa-miR-296-3p | rs6100226 | 20 | 56807638 | T | 0.277 | 0.074 | 3.96E-04 | 0.294 | 0.067 | 0.136 | 6.21E-01 |  |
| hsa-miR-618 | rs2701285 | 12 | 79845482 | C | 0.172 | 0.047 | 4.35E-04 | 0.298 | NA | NA | NA |  |
| hsa-miR-769-5p | rs8108561 | 19 | 51198951 | G | 0.106 | 0.029 | 5.09E-04 | 0.316 | NA | NA | NA |  |
| hsa-miR-618 | rs1716543 | 12 | 79854071 | A | 0.207 | 0.057 | 5.34E-04 | 0.316 | 0.345 | 0.116 | 5.80E-03 |  |
| hsa-miR-146a* | rs2961920 | 5 | 159844084 | C | -0.073 | 0.021 | 5.87E-04 | 0.326 | -0.306 | 0.058 | 6.45E-06 |  |
| hsa-miR-185 | rs4819853 | 22 | 18379760 | G | -0.223 | 0.063 | 6.31E-04 | 0.330 | 0.021 | 0.081 | 1.00E+00 |  |
| hsa-miR-1307 | rs2986014 | 10 | 105184076 | T | 0.204 | 0.057 | 6.90E-04 | 0.341 | 0.124 | 0.117 | 2.96E-01 |  |
| hsa-miR-1303 | rs4958761 | 5 | 154046088 | G | -0.183 | 0.053 | 8.71E-04 | 0.407 | -0.213 | 0.126 | 9.69E-02 |  |
| ^a^miRNA name, ^b^RS identifier for each SNP, ^c^chromosome, ^d^genomics location of SNP, ^e^Effect allele, ^f^coefficient for the SNP effect in the primary study, ^g^standard error for the SNP coefficient in the primary study, ^h^p-value for the SNP effect in the primary study, ^i^FDR adjusted p-value for the SNP effect in the primary study, ^j^coefficient for the SNP effect in the replication study, ^k^standard error for the SNP coefficient in the replication study, ^l^p-value for the SNP effect in the secondary study, ^m^references, (‘NA’ indicate failed assay) | | | | | | | | | | | | |
