# Supplementary material for: MicroRNA Expression in Abdominal and Gluteal Adipose Tissue Is Associated with mRNA Expression Levels and Partly Genetically Driven
Source: PLoS One. 2011 Nov 15;6(11):e27338. doi: 10.1371/journal.pone.0027338 (PMC3216936; doi:10.1371/journal.pone.0027338)
Supplement: Table S5 — Candidate set miRNA eQTLs in gluteal adipose tissue from the primary study together with results from the confirmation study. (DOC) [file pone.0027338.s012.doc]

**Table S5.** Candidate set miRNA eQTLs in gluteal adipose tissue from the primary study together with results from confirmation study.

|  |  |  |  |  | **Primary study** | | | | **Replication study** | | |  |
| --- | --- | --- | --- | --- | --- | --- | --- | --- | --- | --- | --- | --- |
| **miRNA**^a^ | **SNP(rs)**^b^ | **Chr**^c^ | **SNP (Position)**^d^ | **Effect allele**^e^ | **β_snp_**^f^ | **std.error β_snp_**^g^ | **p-value**^h^ | **FDR adjusted p-value**^i^ | **β_snp_ (study2)**^j^ | **std.error β_snp_ (study2)**^k^ | **p-value (study2)**^l^ | **Ref**^m^ |
| hsa-miR-1255a | rs1476159 | 4 | 102500118 | T | 0.138 | 0.029 | 1.56E-05 | 0.069 | 0.175 | 0.033 | 2.89E-05 |  |
| hsa-miR-1255a | rs1822168 | 4 | 102512567 | C | 0.138 | 0.029 | 1.56E-05 | 0.069 | 0.169 | 0.036 | 1.65E-04 |  |
| hsa-miR-30c-1* | rs709688 | 1 | 41025772 | A | -0.748 | 0.178 | 8.65E-05 | 0.256 | 0.194 | 0.728 | 1.00E+00 |  |
| hsa-miR-1307 | rs11191666 | 10 | 105117268 | G | 0.221 | 0.054 | 1.27E-04 | 0.282 | 0.260 | 0.065 | 3.55E-04 |  |
| hsa-miR-330-3p | rs7252175 | 19 | 50825096 | A | -0.198 | 0.053 | 3.85E-04 | 0.595 | -0.338 | 0.160 | 4.04E-02 |  |
| hsa-miR-601 | rs7034379 | 9 | 125172150 | C | 0.053 | 0.014 | 4.66E-04 | 0.595 | NA | NA | NA |  |
| hsa-miR-671-3p | rs7783602 | 7 | 150596099 | T | 0.192 | 0.053 | 4.68E-04 | 0.595 | 0.005 | 0.096 | 9.58E-01 |  |
| hsa-miR-671-3p | rs122815 | 7 | 150571590 | T | 0.290 | 0.081 | 6.64E-04 | 0.606 | NA | NA | NA |  |
| hsa-miR-1255a | rs2583401 | 4 | 102423552 | T | 0.126 | 0.036 | 8.52E-04 | 0.606 | NA | NA | NA |  |
| hsa-miR-214 | rs6694089 | 1 | 170350504 | A | -0.058 | 0.016 | 8.58E-04 | 0.606 | 0.002 | 0.053 | 1.00E+00 |  |
| hsa-miR-9* | rs3431 | 1 | 154640880 | T | 0.206 | 0.059 | 8.74E-04 | 0.606 | -0.103 | 0.053 | 1.00E+00 |  |
| ^a^miRNA name, ^b^RS identifier for each SNP, ^c^chromosome, ^d^genomics location of SNP, ^e^Effect allele, ^f^coefficient for the SNP effect in the primary study, ^g^standard error for the SNP coefficient in the primary study, ^h^p-value for the SNP effect in the primary study, ^i^FDR adjusted p-value for the SNP effect in the primary study, ^j^coefficient for the SNP effect in the replication study, ^k^standard error for the SNP coefficient in the replication study, ^l^p-value for the SNP effect in the secondary study, ^m^references, (‘NA’ indicate failed assay) | | | | | | | | | | | | |
