# Supplementary material for: MicroRNA Expression in Abdominal and Gluteal Adipose Tissue Is Associated with mRNA Expression Levels and Partly Genetically Driven
Source: PLoS One. 2011 Nov 15;6(11):e27338. doi: 10.1371/journal.pone.0027338 (PMC3216936; doi:10.1371/journal.pone.0027338)
Supplement: Table S6 — Genome-wide association analysis results of eQTL SNPs in relation to BMI and waist/hip ratio adjusted for BMI. (DOC) [file pone.0027338.s013.doc]

**Table S6.** Genome-wide association analysis results of eQTL SNPs in relation to BMI and waist/hip ratio adjusted for BMI.

| **Phenotype**^a^ | **MarkerName**^b^ | **P-value**^c^ |
| --- | --- | --- |
| bmi | rs1476159 | 0.48080422 |
| bmi | rs1822168 | 0.44177436 |
| bmi | rs1716543 | 0.49490913 |
| bmi | rs2961920 | 0.31049922 |
| bmi | rs11191666 | 0.26237975 |
| bmi | rs7252175 | 0.06384507 |
| whrAdjBmi | rs1476159 | 0.3736 |
| whrAdjBmi | rs1822168 | 0.3338 |
| whrAdjBmi | rs1716543 | 0.383 |
| whrAdjBmi | rs2961920 | 0.3396 |
| whrAdjBmi | rs11191666 | 0.2375 |
| whrAdjBmi | rs7252175 | 0.9361 |
| ^a^Phenotype tested (bmi = body mass index, whrAdjBmi = Waist – hip ratio adjusted for BMI), ^b^SNP rs identifier, ^c^p-value from association test. | | |
