# Supplementary material for: MicroRNA Expression in Abdominal and Gluteal Adipose Tissue Is Associated with mRNA Expression Levels and Partly Genetically Driven
Source: PLoS One. 2011 Nov 15;6(11):e27338. doi: 10.1371/journal.pone.0027338 (PMC3216936; doi:10.1371/journal.pone.0027338)
Supplement: Table S7 — miRNAs significantly associated with their mRNA targets in abdominal adipose tissue. (DOC) [file pone.0027338.s014.doc]

**Table S7.** miRNAs significantly associated with their mRNA targets in abdominal adipose tissue.

| **miRNA**^a^ | **p.value**^b^ | **p.value.adj**^c^ | **Total number of predicted mRNA targets**^d^ |
| --- | --- | --- | --- |
| hsa-miR-30d | 1.00E-16 | 2.49E-14 | 1007 |
| hsa-miR-181a | 1.21E-14 | 1.50E-12 | 820 |
| hsa-miR-590-3p | 1.05E-09 | 8.65E-08 | 740 |
| hsa-miR-30a | 2.81E-09 | 1.74E-07 | 1007 |
| hsa-miR-302d | 9.77E-09 | 4.85E-07 | 565 |
| hsa-miR-30e | 3.11E-08 | 1.29E-06 | 1007 |
| hsa-miR-124 | 8.12E-08 | 2.81E-06 | 1208 |
| hsa-miR-181c | 9.07E-08 | 2.81E-06 | 820 |
| hsa-miR-543 | 1.13E-07 | 3.13E-06 | 446 |
| hsa-miR-520b,hsa-miR-520c-3p,hsa-miR-520f | 2.01E-06 | 4.89E-05 | 565 |
| hsa-miR-340 | 2.17E-06 | 4.89E-05 | 882 |
| hsa-miR-520c-3p,hsa-miR-520f | 3.79E-06 | 7.83E-05 | 565 |
| hsa-miR-135a | 4.15E-06 | 7.91E-05 | 467 |
| hsa-miR-410 | 4.92E-06 | 8.72E-05 | 392 |
| hsa-miR-186 | 5.54E-06 | 9.16E-05 | 475 |
| hsa-miR-15a | 6.17E-06 | 9.19E-05 | 890 |
| hsa-miR-302b | 6.30E-06 | 9.19E-05 | 565 |
| hsa-miR-32 | 6.69E-06 | 9.21E-05 | 642 |
| hsa-miR-221 | 1.20E-05 | 1.57E-04 | 264 |
| hsa-miR-424 | 1.73E-05 | 2.14E-04 | 890 |
| hsa-miR-363 | 2.42E-05 | 2.86E-04 | 642 |
| hsa-miR-223 | 8.09E-05 | 9.12E-04 | 186 |
| hsa-miR-495 | 9.57E-05 | 1.03E-03 | 523 |
| hsa-miR-141 | 1.02E-04 | 1.05E-03 | 484 |
| hsa-miR-93 | 1.27E-04 | 1.26E-03 | 915 |
| hsa-miR-33b | 2.29E-04 | 2.18E-03 | 243 |
| hsa-miR-454 | 3.24E-04 | 2.96E-03 | 684 |
| hsa-miR-96 | 3.34E-04 | 2.96E-03 | 728 |
| hsa-miR-33a | 3.76E-04 | 3.21E-03 | 243 |
| hsa-miR-520d-3p | 4.79E-04 | 3.96E-03 | 565 |
| hsa-miR-130b | 5.99E-04 | 4.79E-03 | 684 |
| hsa-miR-18b | 7.95E-04 | 6.16E-03 | 175 |
| hsa-miR-182 | 9.66E-04 | 7.26E-03 | 780 |
| hsa-miR-374a | 1.41E-03 | 1.03E-02 | 422 |
| hsa-miR-142-3p | 1.69E-03 | 1.20E-02 | 234 |
| hsa-miR-519d | 1.92E-03 | 1.32E-02 | 915 |
| hsa-miR-372 | 2.76E-03 | 1.85E-02 | 565 |
| hsa-miR-25 | 3.31E-03 | 2.16E-02 | 642 |
| hsa-miR-301a | 3.94E-03 | 2.51E-02 | 684 |
| hsa-miR-429 | 4.68E-03 | 2.87E-02 | 747 |
| hsa-miR-496 | 4.88E-03 | 2.87E-02 | 84 |
| hsa-miR-300 | 4.93E-03 | 2.87E-02 | 595 |
| hsa-miR-133a | 4.97E-03 | 2.87E-02 | 465 |
| hsa-miR-128 | 5.60E-03 | 3.16E-02 | 717 |
| hsa-miR-194 | 6.16E-03 | 3.40E-02 | 234 |
| hsa-miR-144 | 6.53E-03 | 3.52E-02 | 604 |
| hsa-miR-106a | 6.68E-03 | 3.52E-02 | 915 |
| hsa-miR-17 | 7.36E-03 | 3.81E-02 | 915 |
| hsa-miR-140-5p | 8.26E-03 | 4.11E-02 | 231 |
| hsa-miR-183 | 8.29E-03 | 4.11E-02 | 272 |
| hsa-miR-488 | 8.64E-03 | 4.20E-02 | 193 |
| hsa-miR-381 | 9.65E-03 | 4.51E-02 | 595 |
| hsa-miR-425 | 9.81E-03 | 4.51E-02 | 109 |
| hsa-miR-506 | 9.83E-03 | 4.51E-02 | 1208 |
| hsa-miR-137 | 1.09E-02 | 4.93E-02 | 784 |
| ^a^miRNA name, ^b^p-value from gene set enrichment test**,** ^c^FDR adjusted p-value**,** ^d^total number of targets predicted by target scan. | | | |
