# Supplementary material for: MicroRNA Expression in Abdominal and Gluteal Adipose Tissue Is Associated with mRNA Expression Levels and Partly Genetically Driven
Source: PLoS One. 2011 Nov 15;6(11):e27338. doi: 10.1371/journal.pone.0027338 (PMC3216936; doi:10.1371/journal.pone.0027338)
Supplement: Table S8 — miRNAs significantly associated with their mRNA targets in gluteal adipose tissue. (DOC) [file pone.0027338.s015.doc]

**Table S8.** miRNAs significantly associated with their mRNA targets in gluteal adipose tissue.

| **miRNA**^a^ | **p.value**^b^ | **p.value.adj**^c^ | **Total number of predicted mRNA targets**^d^ |
| --- | --- | --- | --- |
| hsa-miR-26a | 2.74E-19 | 6.80E-17 | 593 |
| hsa-miR-145 | 6.47E-08 | 8.02E-06 | 487 |
| hsa-miR-141 | 9.77E-06 | 6.19E-04 | 484 |
| hsa-miR-590-3p | 1.04E-05 | 6.19E-04 | 740 |
| hsa-miR-125a-5p | 1.25E-05 | 6.19E-04 | 555 |
| hsa-miR-27b | 2.03E-05 | 8.38E-04 | 846 |
| hsa-miR-29a | 2.41E-05 | 8.55E-04 | 778 |
| hsa-miR-217 | 3.49E-05 | 1.08E-03 | 212 |
| hsa-miR-128 | 1.14E-04 | 3.14E-03 | 717 |
| hsa-let-7a | 2.35E-04 | 5.82E-03 | 754 |
| hsa-miR-27a | 2.69E-04 | 6.07E-03 | 846 |
| hsa-miR-16 | 4.84E-04 | 1.00E-02 | 890 |
| hsa-miR-28-5p | 1.47E-03 | 2.80E-02 | 98 |
| hsa-miR-30a | 1.73E-03 | 3.07E-02 | 1007 |
| hsa-miR-340 | 2.26E-03 | 3.73E-02 | 882 |
| hsa-miR-181a | 2.47E-03 | 3.83E-02 | 820 |
| hsa-miR-208a | 2.85E-03 | 4.16E-02 | 104 |
| hsa-miR-30d | 3.76E-03 | 4.42E-02 | 1007 |
| hsa-miR-186 | 3.78E-03 | 4.42E-02 | 475 |
| hsa-miR-181b | 3.96E-03 | 4.42E-02 | 820 |
| hsa-miR-196b | 3.96E-03 | 4.42E-02 | 187 |
| hsa-miR-23b | 4.01E-03 | 4.42E-02 | 767 |
| hsa-miR-455-5p | 4.10E-03 | 4.42E-02 | 122 |
| ^a^miRNA name, ^b^p-value from gene set enrichment test**,** ^c^FDR adjusted p-value**,** ^d^total number of targets predicted by target scan. | | | |
