# Supplementary material for: MicroRNA Expression in Abdominal and Gluteal Adipose Tissue Is Associated with mRNA Expression Levels and Partly Genetically Driven
Source: PLoS One. 2011 Nov 15;6(11):e27338. doi: 10.1371/journal.pone.0027338 (PMC3216936; doi:10.1371/journal.pone.0027338)
Supplement: Table S9 — Significant KEGG terms from miRNA-mRNA association analysis in abdominal adipose tissue. (DOC) [file pone.0027338.s016.doc]

**Table S9.** Significant KEGG terms from miRNA-mRNA association analysis in abdominal adipose tissue.

| **kegg pathway**^a^ | **p.value**^b^ | **p.value.adj**^c^ |
| --- | --- | --- |
| TGF-beta signaling pathway | 1.34E-14 | 2.82E-12 |
| Regulation of actin cytoskeleton | 8.22E-13 | 8.63E-11 |
| Endocytosis | 3.17E-12 | 2.21E-10 |
| Chronic myeloid leukemia | 4.21E-12 | 2.21E-10 |
| Neurotrophin signaling pathway | 1.80E-11 | 7.54E-10 |
| Pathways in cancer | 3.79E-11 | 1.22E-09 |
| Prostate cancer | 4.80E-11 | 1.22E-09 |
| mTOR signaling pathway | 4.90E-11 | 1.22E-09 |
| Axon guidance | 5.25E-11 | 1.22E-09 |
| Pancreatic cancer | 3.94E-10 | 8.28E-09 |
| Renal cell carcinoma | 2.05E-09 | 3.92E-08 |
| Glioma | 3.69E-09 | 6.45E-08 |
| Non-small cell lung cancer | 1.16E-08 | 1.88E-07 |
| Focal adhesion | 1.42E-08 | 2.13E-07 |
| Adherens junction | 2.19E-08 | 3.07E-07 |
| ErbB signaling pathway | 3.36E-07 | 4.41E-06 |
| Melanoma | 6.32E-07 | 7.81E-06 |
| Ubiquitin mediated proteolysis | 9.73E-07 | 1.13E-05 |
| Wnt signaling pathway | 1.71E-06 | 1.89E-05 |
| MAPK signaling pathway | 2.47E-06 | 2.59E-05 |
| Long-term potentiation | 3.12E-06 | 3.12E-05 |
| Fc gamma R-mediated phagocytosis | 6.33E-06 | 6.04E-05 |
| Colorectal cancer | 9.96E-06 | 9.09E-05 |
| Circadian rhythm - mammal | 1.55E-05 | 1.35E-04 |
| Acute myeloid leukemia | 4.45E-05 | 3.70E-04 |
| Small cell lung cancer | 4.59E-05 | 3.70E-04 |
| Insulin signaling pathway | 5.61E-05 | 4.37E-04 |
| Progesterone-mediated oocyte maturation | 8.68E-05 | 6.48E-04 |
| Endometrial cancer | 9.16E-05 | 6.48E-04 |
| Dorso-ventral axis formation | 9.26E-05 | 6.48E-04 |
| Aldosterone-regulated sodium reabsorption | 1.25E-04 | 8.49E-04 |
| Gap junction | 1.34E-04 | 8.76E-04 |
| Shigellosis | 2.48E-04 | 1.58E-03 |
| Bacterial invasion of epithelial cells | 3.92E-04 | 2.42E-03 |
| Oocyte meiosis | 5.10E-04 | 3.06E-03 |
| Bladder cancer | 1.13E-03 | 6.60E-03 |
| T cell receptor signaling pathway | 1.76E-03 | 9.99E-03 |
| Type II diabetes mellitus | 1.91E-03 | 1.06E-02 |
| Adipocytokine signaling pathway | 2.54E-03 | 1.37E-02 |
| Phosphatidylinositol signaling system | 5.99E-03 | 3.15E-02 |
| Tight junction | 6.70E-03 | 3.43E-02 |
| Cell cycle | 9.67E-03 | 4.83E-02 |
| Melanogenesis | 9.94E-03 | 4.86E-02 |
| ^a^name of kegg term/pathway, ^b^p-value for enrichment of KEGG term, ^c^FDR adjusted p-value. | | |
