# Supplementary material for: MicroRNA Expression in Abdominal and Gluteal Adipose Tissue Is Associated with mRNA Expression Levels and Partly Genetically Driven
Source: PLoS One. 2011 Nov 15;6(11):e27338. doi: 10.1371/journal.pone.0027338 (PMC3216936; doi:10.1371/journal.pone.0027338)
Supplement: Table S10 — Significant KEGG terms from miRNA-mRNA association analysis in gluteal adipose tissue. (DOC) [file pone.0027338.s017.doc]

**Table S10.** Significant KEGG terms from miRNA-mRNA association analysis in gluteal adipose tissue.

| **kegg pathway**^a^ | **p.value**^b^ | **p.value.adj**^c^ |
| --- | --- | --- |
| Neurotrophin signaling pathway | 1.16E-08 | 2.39E-06 |
| Adherens junction | 2.06E-07 | 1.68E-05 |
| Axon guidance | 2.63E-07 | 1.68E-05 |
| Regulation of actin cytoskeleton | 4.32E-07 | 1.68E-05 |
| Pathways in cancer | 4.63E-07 | 1.68E-05 |
| Chronic myeloid leukemia | 4.89E-07 | 1.68E-05 |
| TGF-beta signaling pathway | 6.48E-07 | 1.91E-05 |
| Focal adhesion | 8.95E-07 | 2.30E-05 |
| Glioma | 1.24E-06 | 2.84E-05 |
| mTOR signaling pathway | 1.91E-06 | 3.94E-05 |
| Prostate cancer | 2.42E-06 | 4.49E-05 |
| Endocytosis | 2.61E-06 | 4.49E-05 |
| Wnt signaling pathway | 3.78E-06 | 5.99E-05 |
| MAPK signaling pathway | 6.25E-06 | 9.19E-05 |
| Pancreatic cancer | 1.12E-05 | 1.54E-04 |
| Renal cell carcinoma | 2.10E-05 | 2.70E-04 |
| ErbB signaling pathway | 2.32E-05 | 2.75E-04 |
| Non-small cell lung cancer | 2.41E-05 | 2.75E-04 |
| T cell receptor signaling pathway | 2.72E-05 | 2.95E-04 |
| Aldosterone-regulated sodium reabsorption | 3.28E-05 | 3.37E-04 |
| Insulin signaling pathway | 1.90E-04 | 1.86E-03 |
| Melanoma | 2.78E-04 | 2.60E-03 |
| Ubiquitin mediated proteolysis | 3.92E-04 | 3.51E-03 |
| Long-term potentiation | 4.31E-04 | 3.70E-03 |
| Fc gamma R-mediated phagocytosis | 4.81E-04 | 3.96E-03 |
| Dorso-ventral axis formation | 6.98E-04 | 5.53E-03 |
| Type II diabetes mellitus | 1.12E-03 | 8.56E-03 |
| Acute myeloid leukemia | 2.07E-03 | 1.52E-02 |
| Oocyte meiosis | 4.00E-03 | 2.84E-02 |
| Small cell lung cancer | 4.45E-03 | 3.06E-02 |
| Colorectal cancer | 4.63E-03 | 3.08E-02 |
| Amyotrophic lateral sclerosis (ALS) | 5.17E-03 | 3.23E-02 |
| Progesterone-mediated oocyte maturation | 5.18E-03 | 3.23E-02 |
| ^a^name of kegg term/pathway, ^b^p-value for enrichment of KEGG term, ^c^FDR adjusted p-value. | | |
