# Supplementary material for: MicroRNA Expression in Abdominal and Gluteal Adipose Tissue Is Associated with mRNA Expression Levels and Partly Genetically Driven
Source: PLoS One. 2011 Nov 15;6(11):e27338. doi: 10.1371/journal.pone.0027338 (PMC3216936; doi:10.1371/journal.pone.0027338)
Supplement: Text S1 — List of MolPAGE consortium partners. (DOC) [file pone.0027338.s018.doc]

# ^#^MolPAGE consortium partners

(In no particular order)

John Bell^1^, Mark McCarthy^1^, Lon Cardon^1^, Peter Donnelly^1^, Edwin Southern**^2^,** John Anson**^2^**, Mark Lathrop**^3^**, Ivo Gut**^3^**, Matthias Schuster**^4^**, Kurt Berlin**^4^**, Esper Boel**^5^**, Jan Fleckner**^5^**, Mathius Uhlen**^6^**, Thomas Bergman**^7^**, Vladimir Stich**^8^**, Alvis Brazma**^9^**, Ugis Sarkans**^9^**, Juris Viksna**^10^**, Jeremy Nicholson**^11^**, Luisa Bernardinelli**^12^**, Stephen Hoffmann**^13^**, Mats Inganas**^13^**, Tim Spector**^14^**, Dominique Langin**^15^**, Fredrik Ponten**^16^** , Hanno Langen**^17^**, Stefan Evers**^17^**

^1^**University of Oxford, Oxford, UK**

**^2^Oxford Gene Technology, Oxford, UK**

**^3^Centre National de Génotypage, Evry, France**

**^4^ Formerly affiliated with Epigenomics AG, Berlin, Germany**

**^5^Novo Nordisk, Copenhagen, Denmark**

**^6^Royal Institute of Technology, Stockholm, Sweden**

**^7^ Formerly affiliated with Affibody AB, Bromma, Sweden**

**^8^Charles University Prague, Czech Republic**

**^9^European Bioinformatics Institute, Cambridge, UK**

**^10^Institute o Mathematics and Computer Science, Riga, Latvia**

**^11^Imperial College of Science, Technology and Medicine, London, UK**

**^12^University of Pavia, Pavia, Italy**

^13^**Gyros AB, Uppsala, Sweden**

**^14^Guys and St Thomas Hospital NHS Trust, London, UK**

**^15^Obesity Research Unit, INSERM, Toulouse, France**

**^16^University of Uppsala, Uppsala, Sweden**

**^17^Roche, Basel, Switzerland**
